# Synthesis, DNA Binding, and Antiproliferative Activity of Novel Acridine-Thiosemicarbazone Derivatives

**DOI:** 10.3390/ijms160613023

**Published:** 2015-06-09

**Authors:** Sinara Mônica Vitalino de Almeida, Elizabeth Almeida Lafayette, Lúcia Patrícia Bezerra Gomes da Silva, Cézar Augusto da Cruz Amorim, Tiago Bento de Oliveira, Ana Lucia Tasca Gois Ruiz, João Ernesto de Carvalho, Ricardo Olímpio de Moura, Eduardo Isidoro Carneiro Beltrão, Maria do Carmo Alves de Lima, Luiz Bezerra de Carvalho Júnior

**Affiliations:** 1 Laboratório de Imunopatologia Keizo Asami (LIKA) and Departamento de Bioquímica, Universidade Federal de Pernambuco (UFPE), Recife 50670-901, PE, Brazil; E-Mails: lucia-patricia@hotmail.com (L.P.B.G.S.); ebeltrao@hotmail.com (E.I.C.B.); lbcj@hotlink.com.br (L.B.C.J.); 2 Faculdade de Ciências, Educação e Tecnologia de Garanhuns (FACETEG), Universidade de Pernambuco (UPE), Garanhuns 55290-000, PE, Brazil; 3 Departamento de Antibióticos, Universidade Federal de Pernambuco (UFPE), Recife 50670-901, PE, Brazil; E-Mails: elizabeth.almeidalafayette@gmail.com (E.A.L.); cezar.amoriim@gmail.com (C.A.C.A.); tiagobento2006@gmail.com (T.B.O.); nenalima.mariadocarmo@gmail.com (M.C.A.L.); 4 Divisão de Farmacologia e Toxicologia, Centro Pluridisciplinar de Pesquisas Químicas, Biológicas e Agrícolas (DFT/CPQBA), Universidade Estadual de Campinas (UNICAMP), Campinas 13083-970, SP, Brazil; E-Mails: aa_ruiz@hotmail.com (A.L.T.G.R.); carvalho@cpqba.unicamp.br (J.E.C.); 5 Faculdade de Ciências Farmacêuticas, Universidade Estadual de Campinas (UNICAMP), Campinas 13083-859, SP, Brazil; 6 Departamento de Farmácia, Laboratório de Síntese e Vetorização de Moléculas, Universidade Estadual da Paraíba (UEPB), Campus Campina Grande 58429-500, PB, Brazil; E-Mail: ricardo.olimpiodemoura@gmail.com

**Keywords:** acridine, thiosemicarbazone, DNA binding, antiproliferative

## Abstract

In this work, the acridine nucleus was used as a lead-compound for structural modification by adding different substituted thiosemicarbazide moieties. Eight new (*Z*)-2-(acridin-9-ylmethylene)-*N*-phenylhydrazinecarbothioamide derivatives (**3a**–**h**) were synthesized, their antiproliferative activities were evaluated, and DNA binding properties were performed with calf thymus DNA (ctDNA) by electronic absorption and fluorescence spectroscopies. Both hyperchromic and hypochromic effects, as well as red or blue shifts were demonstrated by addition of ctDNA to the derivatives. The calculated binding constants ranged from 1.74 × 10^4^ to 1.0 × 10^6^ M^−1^ and quenching constants from −0.2 × 10^4^ to 2.18 × 10^4^ M^−1^ indicating high affinity to ctDNA base pairs. The most efficient compound in binding to ctDNA *in vitro* was (*Z*)-2-(acridin-9-ylmethylene)-*N*-(4-chlorophenyl) hydrazinecarbothioamide (**3f**), while the most active compound in antiproliferative assay was (*Z*)-2-(acridin-9-ylmethylene)-*N*-phenylhydrazinecarbothioamide (**3a**). There was no correlation between DNA-binding and *in vitro* antiproliferative activity, but the results suggest that DNA binding can be involved in the biological activity mechanism. This study may guide the choice of the size and shape of the intercalating part of the ligand and the strategic selection of substituents that increase DNA-binding or antiproliferative properties.

## 1. Introduction

DNA has a strong affinity for many heterocyclic aromatic compounds; hence studies of the interaction between DNA and new chemotherapeutic agents play a key role in the fight against cancer [1]. DNA recognition is a critical step in the antitumor action of DNA intercalators. Given that intercalation constitutes a pivotal step in several clinically used anti-cancer drugs such as anthracyclines, acridines, and anthraquinones [2], understanding how small molecules interact with DNA is crucial for the formulation of more powerful and selective anticancer agents [3].

The synthesis of acridine and analogues has attracted considerable attention from organic and medicinal chemists for many years [4]. Amsacrine (*m*-AMSA) is an anticancer agent that displays activity against refractory acute leukemia as well as Hodgkin and non-Hodgkin’s lymphomas. The drug is comprised of an intercalative acridine moiety coupled to a 4'-amino-methanesulfon-*m*-anisidide head group. *m*-AMSA is historically significant in that it was the first drug demonstrated to function as a topoisomerase II poison. Although *m*-AMSA was designed as a DNA binding agent, much of its activity and specificity as topoisomerase II poison is embodied in the head group, while DNA intercalation is used primarily to increase the affinity of *m*-AMSA for the topoisomerase II-DNA cleavage complex [5]. Furthermore, DNA binding studies indicate that *m*-AMSA binds to DNA both by intercalation and by minor groove binding [6].

Although *m*-AMSA is a DNA-intercalator and inhibitor of topoisomerase II, its metabolism may be associated with the production of free radicals, which causes serious damage in both cancer cells and normal tissues. For this reason, its clinical use is limited and researchers have been seeking to make further chemical modifications to *m*-AMSA by changing the nature of the 9-anilino substituent and the substituent pattern on the aniline and acridine nuclei, leading to a range of new *m*-AMSA-like derivatives [7]. It is worthwhile noting that the replacement of the acridine moiety with the analogous 2-oxo-2H-pyrano [2,3,]quinolone system yielded *m*-AMSA-like compounds that showed drastically reduced anticancer activity as well as an ability to intercalate into double stranded DNA (dsDNA) [8].

One strategy that has been used for the synthesis of new acridine derivatives involves combining the acridine ring with a different and independently acting moiety into one covalently linked hybrid compound, using for example the thiazolidine or imidazolidine nucleus [9]. As Barros *et al.* [10] have demonstrated, synthesizing new acridine-thiazolidine derivatives causes a distortion of the double helix of dsDNA. Concerning the cytotoxicity activity, the thiazacridines were selective for solid tumor cell lines. On the other hand, Lafayette *et al.* [9] provided insight into the DNA binding mechanism of imidazacridine and thiazacridine derivatives suggesting both intercalation and external binding.

Thiosemicarbazone derivatives exhibit important biological activities [11], such as antibacterial [12], antimalarial, [13] and antitumor activities [14]. The antiproliferative properties of thiosemicarbazones have been attributed to their ability to chelate metal ions because of the presence of an NNS (Nitrogen–Nitrogen–Sulfur) tridentate set of donor atoms that bind not only iron, but also copper [15,16,17], molybdenum [18], nickel [16,19], zinc [17,19], and ruthenium [20]. The thiosemicarbazone-metal complexes are lipophilic and pass through the cell membrane to release the metal intracellularly. The free chelator can then complex with intracellular iron that can subsequently pass through the cell membrane mobilizing it out of the cell [21]. Besides, thiosemicarbazone antiproliferative activity can be due to DNA binding and cleavage, apoptosis induction, and cell enzyme inhibition [22,23]. The small molecule chelator 3-aminopyridine-2-carboxaldehyde thiosemicarbazone (3-AP; Triapine^®^, Nanotherapeutics, Alachua, FL, USA) inhibits the ribonucleotide reductase and prevents the replication of tumor cells by blocking a critical step in DNA synthesis [24]. This molecule is under Phase 2 clinical trials evaluation for the treatment of solid tumors such as cervical and vaginal cancers [25].

Recently, several kinds of thiosemicarbazone derivatives were synthesized and their antitumor activities were also reported [26,27,28,29,30]. Geng *et al.* [31] used the anthracyclines as lead-compounds for structural modification by adding thiosemicarbazide to the anthraquinone ring. (*E*)-2-((1,4-Dihydroxy-9,10-anthraquinone-2-yl)methylene)-*N*-(4-fluorophenyl)hydrazinecarbothioamide (DAFPT) was successfully synthesized and its interaction with ctDNA showed groove binding and preference for A–T rich regions. It was expected that this structural modification might overcome drug resistance and decrease the cardiotoxicity of anthracyclines.

In this work, the acridine nucleus was used as a lead-compound for structural modification by adding different substituted thiosemicarbazide moieties. This paper describes the synthesis, DNA binding properties using ctDNA, as well as the antiproliferative activity of novel acridine-thiosemicarbazone derivatives. 

## 2. Results and Discussion

### 2.1. Chemistry

**Scheme 1:**
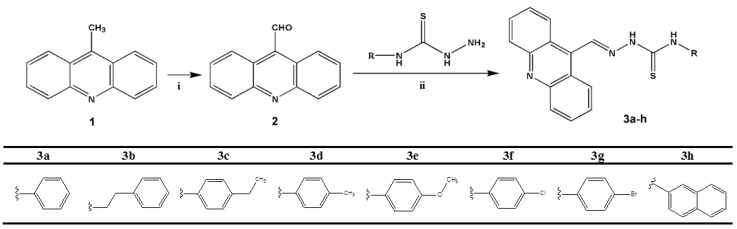
Synthesis of acridine-thiosemicarbazone derivatives. Reagents and conditions: (i) pyridinium chlorochromate (PCC); (ii) EtOH, CH_3_COOH, reflux, 70 °C.

The reaction sequence used for the synthesis of novel acridine-thiosemicarbazone compounds is shown in Scheme 1. The compound 9-methylacridine (**1**) was prepared from diphenylamine with zinc dichloride in acetic acid according to Tsuge *et al.* [32]. Subsequently, the oxidation of **1** with pyridinium chlorochromate (PCC) was accomplished according to Mosher and Natale [33] yielding 9-acridinaldehyde (**2**). Compound **2** was used as starting reagent in the synthesis of different types of acridine-thiosemicarbazone derivatives. Thiosemicarbazides were synthesized in good yields (>90%) by adding hydrazine hydrate to different aryl isothiocyanates as previously described [34]. Thus, condensation of different thiosemicarbazide derivatives with 9-acridinaldehyde afforded the corresponding acridine-thiosemicarbazone derivatives **3a**–**h** (Scheme 1).

All known compounds (**1**, **2** and thiosemicarbazides) were identified by comparison with literature data. All new compounds (**3a**–**h**) were characterized by ^1^H-NMR, ^13^C-NMR, high-resolution mass spectrometry and infrared spectroscopy (Figures S1–S16 and Table S2).

### 2.2. DNA Binding Studies

**Figure 1:**
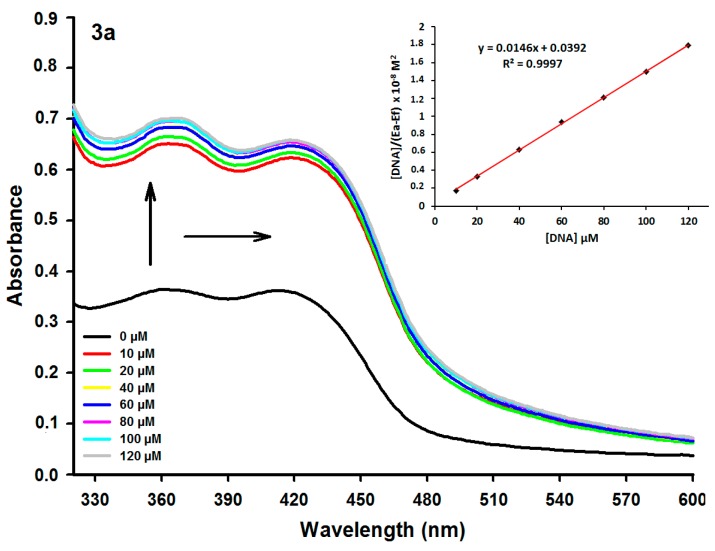
Absorption spectra of derivative **3a** (50 µM) with increasing concentrations of calf thymus DNA (ctDNA). [DNA] = 0, 10, 20, 40, 60, 80, 100 and 120 µM. Arrows (**↑**) and (**→**) refer to hyperchromic, and bathchromic effects, respectively. Inset: Plot of [DNA]/(ε_a_ − ε_f_) as function of DNA concentration as determined from the absorption spectral data.

The absorption spectra of the new acridine-thiosemicarbazone derivatives **3a**–**h** show that all compounds displayed absorption bands in the region of 300–500 nm. The different donor or acceptor substituents present in compounds **3c**–**h** induced red shifts on UV–Vis spectra relative to that of **3a**, with exception of **3f**. In order to examine the general affinity of the derivatives **3a**–**h** to dsDNA, the binding interactions of these ligands with calf thymus DNA (ctDNA) were examined by spectrophotometric titrations. It is widely accepted that if the compounds can bind to DNA, their UV–Vis curves result in a characteristic shift of the absorption maximum wavelength (bathochromic or hypsochromic shift) and a decrease (hypochromicity) [35,36] or increase (hyperchromicity) of the absorbance [9]. The absorption spectra of **3a** (50 µM) in both the absence and presence of ctDNA (0–120 µM) are given in Figure 1 (for the other derivatives see Figures S17–S23). The curve shows significant hyperchromicity (92.58% at 120 µM of ctDNA) and a slight bathochromic shift (Δλ 6 nm) with increasing ctDNA concentration, indicating a complex **3a**-DNA formation. In the presence of ctDNA, all compounds (**3a**–**h**) produced hyperchromic or hypochromic effects, with the highest hypochromism being induced by the **3f** derivative (Table 1). With the exception of compound **3g**, all complexes with ctDNA showed bathochromic or hypsochromic effects with the most remarkable shifts for derivatives **3a** and **3h**, 6 and 12 nm, respectively.

**Table 1:** UV–Vis absorption and fluorescence emission data of new acridine-thiosemicarbazone derivatives in the absence and presence of ctDNA.

Compound	λ_max_ Absent (nm)	λ_max_ Present (nm)	Extinction Coefficient (ε, M^−1^)	Δλ (nm)	Hypochr. (%) ^a^	Hyperchr. (%) ^b^	K_b_ (M^−1^)	λ Excitation (nm)	λ Emission (nm)	K_SV_^c^ (M^−1^)
**3a**	360	366	7.300	6	–	92.58	3.77 × 10^5^	359	439	0.92 × 10^4^
**3b**	375	372	8.600	3	–	28.13	1.84 × 10^5^	370	441	−0.2 × 10^4^
**3c**	376	375	13.780	1	20.32	–	1.2 × 10^5^	370	440	0.18 × 10^4^
**3d**	375	371	14.040	4	4.69	–	1.74 × 10^4^	370	441	0.87 × 10^4^
**3e**	375	371	8.760	4	–	25.22	6.5 × 10^4^	361	441	−0.27 × 10^4^
**3f**	357	358	14.860	1	39.59	–	1.0 × 10^6^	355	440	2.18 × 10^4^
**3g**	362	362	9.200	0	–	24.4	9.91 × 10^4^	352	439	0.5 × 10^4^
**3h**	383	371	11.546	12	11.54	–	8.47 × 10^5^	350	439	1.75 × 10^4^

^a^ Hypochromicity for complexes formed by compounds (**3a–h**) and 120 µM of ctDNA in comparison to free ligands; ^b^ Hyperchromicity for complexes formed by compounds (**3a–h**) and 120 µM of ctDNA in comparison to free ligands; ^c^ Stern–Volmer quenching constant, K_SV_, obtained from fluorimetric titrations with ctDNA.

After compound intercalation into the DNA base pairs, the π orbitals of the intercalated compounds are able to couple with the π orbitals of the base pairs, thereby decreasing the π → π* transition energies [15]. These interactions result in the observed hypochromism that is predictive of intercalation [37]. Besides this, the hypochromic effect and the red shift of the absorption maximum are characteristic of the association of a ligand with DNA [38] which is similar to the interaction recently observed for ctDNA and thiazacridine [9]. These results indicate that the acridine ring has an important role in the DNA interaction process [8] and confirm that both hyperchromic and hypochromic effects, as well as red or blue shifts reflect different degrees of DNA helix structure changes [39].

Absorbance intensity changes were used to calculate the DNA binding constants (K_b_) of the acridine-thiosemicarbazone derivatives according to McGhee and von Hippel (Table 1) [40]. The calculated K_b_ ranged from 1.74 × 10^4^ to 1.0 × 10^6^ M^−1^, proves a high affinity of the new acridine-thiosemicarbazone derivatives for ctDNA base pairs. Typical K_b_ for intercalation complexes between organic dyes and DNA range from 1 × 10^4^ to 1 × 10^6^ M^−1^ and are usually significantly smaller than the binding constants of groove binders (1 × 10^5^ to 1 × 10^9^ M^−1^) [41]. The K_b_ values increased as follows: **3d** < **3e** < **3g** < **3c** < **3b** < **3a** < **3h** < **3f**. The high K_b_ presented by **3f** suggests a strong binding towards ctDNA.

Acridine substitution with thiosemicarbazone moiety influenced positively the intercalation power of the new derivatives since amsacrine-DNA link analysis has shown that the binding constant of the formed complex was K_b_ = 1.2 × 10^4^ M^−1^, indicating weak or moderate strength of binding between them [6]. The binding constants of 3,6-bis(3-alkylguanidino)acridines with ctDNA investigated by UV–Vis and fluorescence spectroscopies, were estimated to range from 1.25 × 10^5^ to 5.26 × 10^5^ M^−1^ and the percentage of hypochromism was found to be 17%–42% (from spectral titration) [42]. DNA binding constant studies of *N*-(9-acridinylthiocarbamoyl) amino acids (glycine, proline, leucine, histidine, phenylalanine, and tryptophan) derivatives by UV–Vis spectrophotometry, fluorescence titration, and quantum chemical calculation revealed that the lowest binding affinity was observed for *N*-(9-acridinylthiocarbamoyl)glycine (K = 1.4 × 10^5^) and the highest for *N*-(9-acridinylthiocarbamoyl)tryptophan (K = 2.9 × 10^6^) [43].

Herein, the highest K_b_ value (1.0 × 10^6^) was demonstrated by the chloro-substituted derivative **3f**. This result indicates that a chloro substituent affects positively the ability of a ligand to bind to ctDNA, since it can increase the ligand’s hydrophobic properties. In addition, the chloro atom leads to a slightly changed dipole of the ligand, thus increasing dipole–dipole interactions in the binding site, and it may even operate as a hydrogen-bond acceptor [44]. Derivative **3h** presented the second highest K_b_ value (8.47 × 10^5^), this finding supports the idea that intercalative π-stacking interactions of the ligands with DNA are essential for their efficient electron transfer reaction. Moreover, uniformity of K_b_ magnitudes for different substituted acridine rings validates the assumption that the DNA binding mechanism is heavily dependent on a common structural component of all studied systems, that is, the acridine skeleton, while its lateral substituents influence binding to a much lesser extent [45].

**Figure 2:**
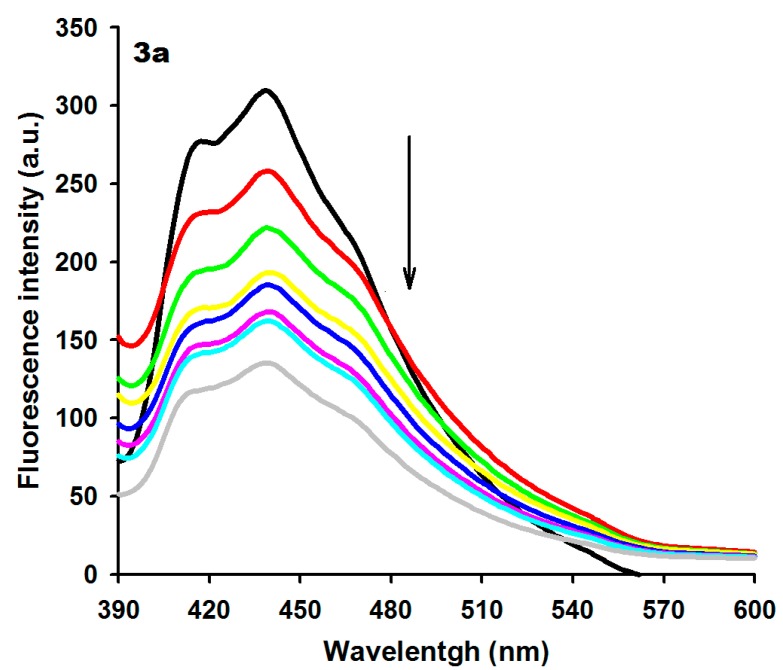
Fluorescence spectra of derivative **3a** (15 µM) with increasing concentrations of ctDNA. [DNA] = 0 (black), 10 (red), 20 (green), 40 (yellow), 60 (blue), 80 (pink), 100 (light blue) and 120 (gray) µM. Arrow (↓) refers to hypochromic effect.

Spectrofluorimetric studies were also performed to analyze the binding properties between acridine-thiosemicarbazone derivatives and ctDNA. Table 1 summarizes the fluorescence emission from the derivatives under investigation (excitation and emission spectra can be seen in Figures S24–S31). Compounds **3a**–**h** exhibited an emission band in the range of 400–500 nm. Excitation wavelengths were at 350–370 nm and spectra were monitored at a fixed concentration of 15 µM of each derivative and different ctDNA concentrations. The fluorescence of acridine-thiosemicarbazone derivatives was quenched upon addition of ctDNA, except for **3b** and **3e** derivatives that exhibited an increase in their fluorescence intensity upon DNA addition. In general, the enhancement of fluorescence intensity may be explained by a significant suppression of the conformational flexibility of the ligand within the DNA–ligand complex [38]. Figure 2 presents the emission spectra of **3a** in the presence of different concentrations of ctDNA (for the other derivatives see Figures S32–S38).

**Figure 3:**
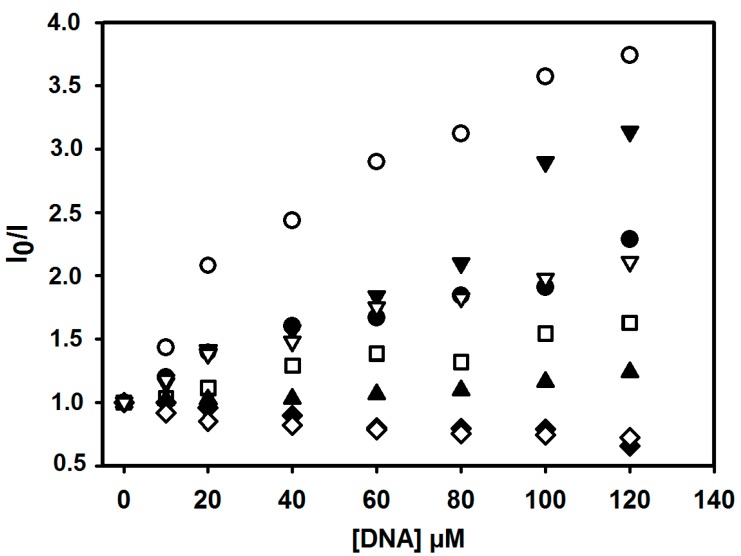
Relative fluorescence intensities of acridine-thiosemicarbazone derivatives **3a** (●), **3b** (◊), **3c** (▲), **3d** (▽), **3e** (♦), **3f** (○), **3g** (□) and **3h** (▼), upon addition of ctDNA in Tris buffer (0.01 M, pH = 7.6).

The efficiency of the quenching process was deduced from the plot of the relative emission intensity, I_0_/I, *vs.* the DNA concentration (Figure 3) [46]. According to the resulting Stern–Volmer quenching constants, K_SV_, the most pronounced quenching was demonstrated by **3f** derivative (2.18 × 10^4^ M^−1^). K_SV_ values decreased as follows: **3f** > **3h** > **3a** > **3d** > **3g** > **3c** > **3e** > **3b**. The emission intensity of the derivatives is quenched upon addition of DNA, most likely due to an efficient electron transfer between the excited ligand and the ctDNA bases. Emission-quenching phenomena reflect the interaction between the derivatives and ctDNA, consistent with the electronic absorption spectroscopy results [47]. Both Stern–Volmer (K_SV_) and binding constants (K_b_) of the derivatives indicate static quenching due to complex formation by the new derivatives and ctDNA. The results indicate that the most efficient compound in binding to ctDNA *in vitro* was **3f** (39.59% hypochromism and the highest K_b_, K_SV_ values).

According to fluorescence emission studies of the interaction of ctDNA and 3ʹ-azido-daunorubicin thiosemicarbazone performed by Cui *et al.* [48], the increasing amounts of ctDNA caused fluorescence intensity decrease without apparent shift of maximum fluorescence emission wavelength, which indicated the ctDNA binding. Interaction evaluation of DAFPT and ctDNA by competitive DNA binding assay demonstrated that DAFPT induced a fluorescence intensity decrease of ctDNA-ethidium bromide complex, probably through displacement of intercalated ethidium bromide from the adjacent base pairs of ctDNA [31]. These results demonstrated that thiosemicarbazone groups do not impair the DNA binding properties of other well established intercalators such as daunorubicin and the anthraquinone ring. In this work, the new acridine-thiosemicarbazones were able to interact with ctDNA since both the “light up” and “light off” effects were observed in fluorescence emission spectra.

### 2.3. Antiproliferative Activity of **3a**–**h**

To determine the antiproliferative properties of the derivatives **3a**–**h**, their *in vitro* antitumoral activities were assessed using nine different human tumor cell lines: glioma (U251), leukemia (K-562), breast (MCF7), breast resistant (NCI-ADR), kidney (786-O), lung (NCI-460), ovarian (OVCAR), prostate (PC-3), and colon (HT-29) and a normal cell line of human keratinocyte (HaCat). The compounds were used at concentrations ranging from 0.25 to 250 mg/mL; doxorubicin (DOX) and *m*-AMSA were used as positive controls. The analysis of cell growth inhibition (GI) was performed using the sulforhodamine B (SRB) assay. The anti-cancer activity (µM) of a tested compound was given by two parameters for each cell line: GI_50_ (molar concentration of the compound that inhibits 50% cell growth) and TGI (molar concentration of the compound leading to total inhibition of the cell growth) [49].

**Figure 4:**
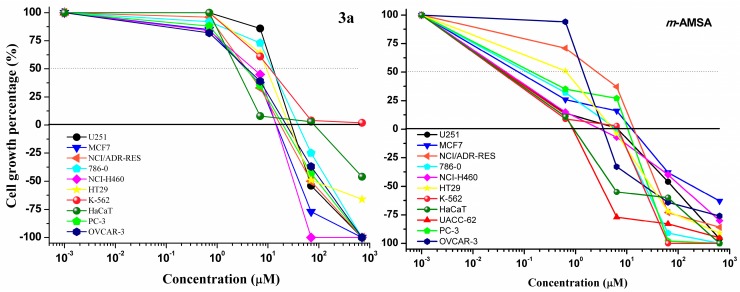
Antiproliferative activity of **3a** (concentrations of 0.7, 7.0, 70 and 702 µM) and amsacrine (*m*-AMSA)(concentrations of 0.6, 6.35, 63.5 and 635 µM) against nine cancerous cell lines: U251 (glioma, SNC); MCF-7 (breast adenocarcinoma); NCI-ADR/RES (ovary, multidrug resistance phenotype); 786-O (kidney); NCI-H460 (lung non-small cell adenocarcinoma); PC-3 (prostate); OVCAR-3 (ovary); HT-29 (colon); K-562 (Chronic myeloid leukemia) and human keratinocytes (HaCaT).

The type of side chain influences the cytotoxic activities of acridine derivatives [50]. For acridine-thiosemicarbazone derivatives, the nature of the substituent on the phenyl ring significantly influenced the antiproliferative activity (Table 2). The most active derivative was **3a** as demonstrated in Figure 4 (for the other derivatives see Figures S39–S45). Compound **3a** inhibited the growth of all cell lines in a dose-dependent manner, with GI_50_ values lower than 10 µM for all tumor cell lines. Tumor cell selectivity was not observed in the growth inhibition, but the pattern was similar to the positive control *m*-AMSA, also presented in Figure 4. However, the GI_50_ concentrations of **3a** ranged widely from 3- to 30-fold higher than those for *m*-AMSA.

Notably, derivatives with electron-withdrawing (**3f**, **3g**) and electron-donating substituents (**3c**, **3d** and **3e**) presented a dramatic decrease in antiproliferative activity as demonstrated by GI_50_ and TGI values (Table 2). Compound **3b** bears an ethylene group as spacer between the nitrogen of the thiosemicarbazone moiety (acridine–C=N–N–C–(S)–N) and the phenyl ring that negatively influenced the antiproliferative activity when compared to **3a**, as indicated by the difference in GI_50_ values (approximately 10-times higher). It was supposed that a high rotational freedom for the phenyl ring in **3b** permitted substituent rotation negatively influencing its growth inhibition activity. Bulky substituents on the phenyl ring also caused a decrease of antiproliferative power, as can be seen from the GI_50_ and TGI values of the pair **3a** and **3h**. Derivative **3g** presenting the 4-bromophenyl moiety exhibited selective cytostatic effects on OVCAR-3 cells.

The lethal concentration LC_50_, represents the drug concentration required to kill 50% of the initial cell number, giving an idea of the cytotoxic action of the drug. Considering the LC_50_ (50% cell kill) values, compound **3a** was lethal to NCI-H460, MCF-7, U251, NCI-ADR/RES, HT-29, and PC-3 cells at the respective concentrations: 43.41, 60.26, 68.93, 70.2, 70.24, and 72.95 µM. For the others cell lines, **3a** LC_50_ concentrations were above 300 µM.

**Table 2:** GI_50_ and TGI (in µM) values for tested compounds **3a**–**h**, doxorubicin (DOX) and amsacrine (*m*-AMSA) as positive controls.

Compound	Cancer Cell Lines ^a^																			
	U251		MCF-7		NCI-ADR		786-O		NCI-H460		PC-3		OVCAR-3		HT-29		K-562		HaCaT	
	GI_50_	TGI	GI_50_	TGI	GI_50_	TGI	GI_50_	TGI	GI_50_	TGI	GI_50_	TGI	GI_50_	TGI	GI_50_	TGI	GI_50_	TGI	GI_50_	TGI
**3a**	8.74	33.75	6.85	12.92	6.35	17.24	7.99	41.66	6.97	10.58	6.30	18.88	4.41	21.13	7.54	29.28	9.26	>100	4.17	51.42
**3b**	80.84	>100	66.72	n.a.	>100	n.a.	64.44	92.31	16.75	>100	73.74	>100	7.26	98.48	>100	n.a.	n.a.	n.a.	>100	n.a.
**3c**	>100	n.a.	62.73	n.a.	62.94	n.a.	28.75	>100	8.63	n.a.	22.98	n.a.	8.38	>100	n.a.	n.a.	n.a.	n.a.	7.00	>100
**3d**	n.a.	n.a.	n.a.	n.a.	n.a.	n.a.	n.a.	n.a.	n.a.	n.a.	n.a.	n.a.	n.a.	n.a.	n.a.	n.a.	n.a.	n.a.	n.a.	n.a.
**3e**	n.a.	n.a.	n.a.	n.a.	n.a.	n.a.	n.a.	n.a.	n.a.	n.a.	n.a.	n.a.	n.a.	n.a.	n.a.	n.a.	n.a.	n.a.	n.a.	n.a.
**3f**	>100	n.a.	27.19	>100	73.15	>100	>100	n.a.	28.36	n.a.	40.33	n.a.	62.41	>100	>100	n.a.	64.64	>100	>100	n.a.
**3g**	>100	n.a.	n.a.	n.a.	n.a.	n.a.	>100	n.a.	>100	n.a.	45.89	>100	0.98	65.7	n.a.	n.a.	n.a.	n.a.	n.a.	n.a.
**3h**	>100	n.a.	69.24	n.a.	68.26	>100	>100	n.a.	70.37	n.a.	50.88	n.a.	71.39	>100	n.a.	n.a.	n.a.	n.a.	70.06	n.a.
DOX ^b^	0.046	1.20	0.036	1.48	0.24	3.80	0.051	0.63	0.04	0.54	0.075	1.20	0.17	1.52	0.43	8.37	0.18	27.64	0.053	0.42
*m*-AMSA	0.26	2.81	0.23	6.96	2.01	11.94	0.52	3.14	0.47	2.58	0.39	5.37	0.87	4.26	0.64	4.66	0.18	1.82	0.46	0.86

^a^ U251 (glioma, SNC); MCF-7 (breast adenocarcinoma); NCI-ADR/RES (ovary, multidrug resistance phenotype); 786-O (kidney); NCI-H460 (lung non-small cell adenocarcinoma); PC-3 (prostate); OVCAR-3 (ovary); HT-29 (colon); K-562 (Chronic myeloid leukemia); HaCaT (human keratinocytes); ^b^ Doxorubicin (DOX) was employed as the positive control. Cell lines were exposed to the compounds in DMSO/RPMI/FBS 5% at 37 °C, 5% CO_2_, for 48 h. n.a. = non-active at highest tested concentration (600 µM).

Even though there was no quantitative correlation between DNA-binding and *in vitro* activity, a lack of activity was observed for **3d** and **3e**, which possess the weakest K_b_ values, 1.74 × 10^4^ and 6.5 × 10^4^, respectively, and the most active compound **3a** presented the third highest K_b_ value (3.77 × 10^5^). The chloro-substituted derivative **3f** showed the highest K_b_ value, but its antiproliferative activity was dramatically lower than **3a** and similar to other substituent groups that have essentially the same steric requirement such as methyl- and bromo-substituted derivatives, **3d** and **3g** respectively. Our findings suggest that the biological activity could be due to the interaction with DNA and/or to any other not studied mechanism. This study may guide the choice of the size and shape of the intercalating part of the ligand and the strategic selection of substituents that increase DNA-binding or antiproliferative properties.

## 3. Experimental Section

### 3.1. Materials and Methods

The starting materials hydrazine hydrate, phenyl-, 4-tolyl-, 4-chlorophenyl-, 1-naphthyl-, 4-bromophenyl-, 4-methoxyphenyl-, 4-ethylphenyl- and 2-phenylethyl-isothiocyanate were purchased from Sigma-Aldrich (Saint Louis, MO, USA) and were used as received for the synthesis of different thiosemicarbazides. Common solvents used for synthesis and analysis were supplied by Sigma-Aldrich (Saint Louis, MO, USA), Merck (Darmstadt, Germany) and Fluka (Buchs, SG, Switzerland) and used without purification. Melting points were measured in capillary tubes on a Quimis Model 340.27 apparatus (Quimis, Diadema, Brasil). Thin Layer Cromatography (TLC) was performed on silica gel 60 F254 plates from Merck (Darmstadt, Germany) with fluorescent detection at 254 nm. Infrared (IR) spectroscopy were recorded on a Bruker IFS66 spectrometer (Bruker, Berlin, Germany), using KBr pellets. The proton (^1^H-NMR) and carbon (^13^C-NMR) NMR experiments were performed on Varian Model Plus Spectrophotometer (Varian, Santa Clara, CA, USA) at 300 MHz, in DMSO-*d*_6_ as solvent. Mass spectra were recorded on matrix-assisted laser desorption/ionization recorded with a time-of-flight mass spectrometer (MALDI-TOF) Autoflex III (Bruker Daltonics, Billerica, MA, USA). UV–Vis spectra were measured on an Ultraspec 3000 PRO UV–Visible spectrophotometer (Biochrom Ltd., Cambridge, UK) and fluorescence spectra on a JASCO FP-6300 (Jasco Corporation, Tokyo, Japan) spectrofluorometer.

### 3.2. General Preparation of the Acridine-Thiosemicarbazone Derivatives **3a**–**h**

The starting thiosemicarbazide (*N*-phenylhydrazinecarbothioamide; *N*-(4-chlorophenyl)hydrazinecarbothioamide; *N*-(naphtalen-1-yl)hydrazinecarbothioamide; *N*-(4-bromophenyl)hydrazinecarbothioamide; *N*-(4-methoxyphenyl)hydrazinecarbothioamide; *N*-phenethylhydrazinecarbothioamide; *N*-(4-ethylphenyl)hydrazinecarbothioamide and *N*-(*p*-tolyl)hydrazinecarbothioamide) were prepared according to the published procedures [34,51]. Compound **2**, 9-acridinaldehyde, (1.0 equivalent) and the thiosemicarbazide of choice (1.0 equivalent), were refluxed in ethanol (10 mL) in the presence of acetic acid (30 drops) and heated at 70 °C for 24 h. After this period, the mixture was filtered and washed with water and ethanol.

#### 3.2.1. (*Z*)-2-(Acridin-9-ylmethylene)-*N*-phenylhydrazinecarbothioamide (**3a**)

Yellow solid. Formula: C_21_H_16_N_4_S; M.W.: 356.44 g/mol; Melting point: 208–209 °C; Yield: 65%; Rf: 0.55 (*n*-hexane/EtOAc 7:3). ^1^H-NMR (300 MHz, DMSO-*d*_6_): δ 7.19 (t, 1H, phenyl, *J* = 7.5 Hz), 7.36 (t, 2H, phenyl, *J* = 7.8 Hz), 7.60 (d, 2H, phenyl, *J* = 7.8 Hz), 7.72 (t, 2H, acridine, *J* = 8.4 Hz), 7.89 (t, 2H, acridine, *J* = 8.4 Hz), 8.20 (d, 2H, acridine, *J* = 8.7 Hz), 8.61 (d, 2H, acridine, *J* = 8.7 Hz), 9.39 (s, 1H, =CH), 10.23 (s, 1H, NH), 12.27 (s, 1H, NH). ^13^C-NMR: δ 123.3, 123.9, 124.9, 125.1, 126.9, 127.9, 128.4, 129.5, 130.8, 130.2, 134.6, 138.8, 139.8, 148.1, 176.3. IR (cm^−1^): 3114 (C–H), 3500 (NH), 1202 (C=S). MS (*m*/*z*): calculated = 357.1129; found = 357.124.

#### 3.2.2. (*Z*)-2-(Acridin-9-ylmethylene)-*N*-phenethylhydrazinecarbothioamide (**3b**)

Yellow solid. Formula: C_23_H_20_N_4_S; M.W.: 384.49 g/mol; Melting point: 220–221 °C; Yield: 97%; Rf: 0.48 (*n*-hexane/EtOAc 8:2). ^1^H-NMR (400 MHz, DMSO-*d*_6_): δ 2.93 (t, 3H, *J* = 10.4 Hz, CH_3_), 3.80 (q, 2H, *J* = 8.4 Hz, CH_2_), 7.27–7.18 (m, 5H, phenyl), 7.68 (t, 2H, *J* = 10.0 Hz, acridine), 7.88 (t, 2H, *J* = 9.6 Hz, acridine), 8.19 (d, 2H, *J* = 11.6 Hz, acridine), 8.49 (d, 2H, *J* = 10.8 Hz, acridine), 8.50 (s, 1H, =CH–), 9.27 (s, 1H, NH), 11.86 (s, 1H, NH). ^13^C-NMR: δ 34.92, 45.67, 123.88, 125.66, 125.66, 126.64, 126.64, 127.59, 127.59, 128.88, 128.88, 129.12, 129.12, 130.18, 130.18, 130.76, 130.76, 135.48, 139.56, 139.68, 148.71, 148.71, 177.77. IR (cm^−1^): 3667.95 (NH), 3140.25 (NH), 1520.45 (C=S). MS (*m*/*z*): calculated = 385.1442; found = 385.134.

#### 3.2.3. (*Z*)-2-(Acridin-9-ylmethylene)-*N*-(4-ethylphenyl)hydrazinecarbothioamide (**3c**)

Pale green solid. Formula: C_23_H_20_N_4_S; M.W.: 384.49 g/mol; Melting point: 213–214 °C; Yield: 98%; Rf: 0.6 (*n*-hexane/EtOAc 7:3). ^1^H-NMR (400 MHz, DMSO-*d*_6_): δ 1.18 (t, 3H, *J* = 7.2 Hz, CH_3_), 2.57 (q, 2H, *J* = 7.2 Hz, CH_2_), 7.18 (d, 2H, *J* = 8.4 Hz, acridine), 7.48 (d, 2H, *J* = 8.4 Hz, acridine), 7.71 (t, 2H, *J* = 7.2 Hz, acridine), 7.89 (t, 2H, *J* = 6.8 Hz, acridine), 8.20 (d, 2H, *J* = 8.8 Hz, phenyl), 8.60 (d, 2H, *J* = 8.4 Hz, phenyl), 9.38 (s, 1H, =CH–), 10.08 (s, 1H, NH), 12.16 (s, 1H, NH). ^13^C-NMR: δ 15.66, 27.70, 123.47, 123.47, 125.33, 125.33, 125.40, 125.40, 127.16, 127.16, 127.38, 127.38, 129.67, 129.67, 130.32, 130.32, 134.86, 136.57, 139.81, 140.91, 148.23, 148.23, 176.43. IR (cm^−1^): 1536.91 (C=S), 2968.91 (=CH–), 3125.53 (NH), 3400.00 (NH). MS (*m*/*z*): calculated = 385.1442; found = 385*.*131.

#### 3.2.4. (*Z*)-2-(Acridin-9-ylmethylene)-*N*-(p-tolyl)hydrazinecarbothioamide (**3d**)

Yellow solid. Formula: C_22_H_18_N_4_S; M.W.: 370.47 g/mol; Melting point: 119–221 °C; Yield: 90%; Rf: 0.55 (*n*-hexane/EtOAc 7:3). ^1^H-NMR (400 MHz, DMSO-*d*_6_): δ 2.29 (s, 3H, CH_3_), 7.15 (d, 2H, *J* = 8.4 Hz, acridine), 7.45 (d, 2H, *J* = 7.6 Hz, acridine), 7.71 (t, 2H, *J* = 7.6 Hz, acridine), 7.89 (t, 2H, *J* = 7.6 Hz, acridine), 8.20 (d, 2H, *J* = 9.2 Hz, phenyl), 8.60 (d, 2H, *J* = 8.4 Hz, phenyl), 9.38 (s, 1H, = CH–), 10.01 (s, 1H, NH), 12.15 (s, 1H, NH). ^13^C-NMR: δ 20.56, 123.48, 123.48, 125.34, 125.34, 125.37, 125.37, 127.16, 127.16, 128.56, 128.56, 129.68, 129.68, 130.32, 130.32, 134.55, 134.89, 136.37, 139.80, 148.25, 176.48. IR (cm^−1^): 3671.99 (NH), 3129.28 (NH), 1534.92 (C=S). MS (*m*/*z*): calculated = 371.1286; found = 371*.*120.

#### 3.2.5. (*Z*)-2-(Acridin-9-ylmethylene)-*N*-(4-methoxyphenyl)hydrazinecarbothioamide (**3e**)

Yellow solid. Formula: C_22_H_18_N_4_OS; M.W.: 386.47 g/mol; Melting point: 219–220 °C; Yield: 82%; Rf: 0.43 (*n*-hexane/EtOAc 7:3). ^1^H-NMR (400 MHz, DMSO-*d*_6_): δ 3.76 (s, 3H, OCH_3_), 6.91 (m, 2H, acridine), 7.46 (m, 2H, acridine), 7.70 (m, 2H, phenyl), 7.87 (m, 2H, phenyl), 8.19 (m, 2H, acridine), 8.60 (m, 2H, acridine), 9.38 (s, 1H, =CH–), 9.25 (s, 1H, NH), 12.02 (s, 1H, NH). ^13^C-NMR: δ 55.09, 113.21, 113.21, 123.32, 123.32, 125.09, 125.09, 126.69, 126.69, 126.86, 126.86, 129.50, 129.50, 129.99, 129.99, 131.69, 134.66, 139.57, 148.11, 148.11, 156.83, 176.67. IR (cm^−1^): 1514.52 (C=N), 1533.44 (C=S), 3138.17 (NH), 3320.32 (NH). MS (*m*/*z*): calculated = 387.1235; found = 387*.*073.

#### 3.2.6. (*Z*)-2-(Acridin-9-ylmethylene)-*N*-(4-chlorophenyl)hydrazinecarbothioamide (**3f**)

Yellow solid. Formula: C_21_H_15_ClN_4_S; M.W.: 390.88 g/mol; Melting point: 225–226 °C; Yield: 86.5%; Rf: 0.54 (*n*-hexane/EtOAc 7:3). ^1^H-NMR (400 MHz, DMSO-*d*_6_): 7,40 (d, 2H, phenyl, *J* = 6.8 Hz), 7.63 (d, 2H, phenyl, *J* = 7.6 Hz), 7.72 (t, 2H, acridine, *J* = 7.2 Hz), 7.89 (t, 2H, acridine, *J* = 7.2 Hz), 8.21 (d, 2H, acridine, *J* = 9.2 Hz), 8.59 (d, 2H, acridine, *J* = 8.4 Hz), 9.39 (s, 1H, =CH–), 10.23 (s, 1H, NH), 12.27 (s, 1H, NH). ^13^C-NMR: δ 123.3, 123.9, 124.9, 125.1, 126.9, 127.9, 128.4, 129.5, 130.8, 130.2, 134.6, 138.8, 139.8, 148.1, 176.3. IR (cm^−1^): 3114 (C–H), 3500 (NH), 1202 (C=S). MS (*m*/*z*): calculated = 391.0739; found = 391*.*041.

#### 3.2.7. (*Z*)-2-(Acridin-9-ylmethylene)-*N*-(4-bromophenyl)hydrazinecarbothioamide (**3g**)

Yellow solid. Formula: C_21_H_15_BrN_4_S; M.W.: 435.34 g/mol; Melting point: 222–224 °C; Yield: 82%; Rf: 0.45 (*n*-hexane/EtOAc 7:3). ^1^H-NMR (300 MHz, DMSO-*d*_6_): δ 7.56 (q, 4H, phenyl, *J* = 8.8 Hz), 7.75 (t, 2H, *J* = 8.4 Hz, acridine), 7.94 (t, 2H, *J* = 8.0 Hz, acridine), 8.23 (d, 2H, *J* = 8.8 Hz, acridine), 8.62 (d, 2H, *J* = 8.8 Hz, acridine), 9.40 (s, 1H, =CH–), 10.24 (s, 1H, NH), 12.31 (s, 1H, NH). ^13^C-NMR: δ 117.60, 117.60, 123.52, 123.52, 123.52, 123.52, 125.51, 125.51, 127.34, 127.34, 127.45, 127.45, 127.45, 127.45, 130.91, 130.91, 130.91, 138.38, 140.05, 140.05, 176.48. IR (cm^−1^): 3350.00 (NH), 3128.54 (NH), 1539.02 (C=S). MS (*m*/*z*): calculated = 436.0180; found = 436*.*968.

#### 3.2.8. (*Z*)-2-(Acridin-9-ylmethylene)-*N*-(naphtalen-1-yl)hydrazinecarbothioamide (**3h**)

Yellow solid. Formula: C_25_H_18_N_4_S; M.W.: 406.50 g/mol; Melting point: 208–210 °C; Yield: 32%; Rf: 0.55 (*n*-hexane/EtOAc 6:4). ^1^H-NMR (300 MHz, DMSO-*d*_6_): δ 7.49 (t, 2H, *J* = 8.1 Hz, acridine), 7.58–7.54 (m, 3H, naphthyl), 7.61 (t, 2H, *J* = 8.4 Hz, acridine), 7.78–8.50 (m, 3H, naphthyl), 8.17 (d, 2H, *J* = 7.8 Hz, acridine), 8.28–8.32 (m, 1H, naphthyl), 8.82 (d, 2H, *J* = 8.7 Hz, acridine), 9.51 (s, 1H, =CH–), 10.49 (s, 1H, NH), 12.41 (s, 1H, NH). ^13^C-NMR: δ 157.95, 136.69, 133.85, 133.85, 133.85, 128.29, 128.29, 127.59, 126.04, 126.04, 126.04, 126.04, 125.63, 125.63, 125.63, 125.27, 125.27, 125.27, 123.56, 123.56, 122.69, 122.69, 121.91, 121.91, 115.37. IR (cm^−1^): 3309.48 (NH), 3199.93 (NH), 1471.72 (C=S), 1212.81 (C–N). MS (*m*/*z*): calculated = 407.1286; found = 407*.*024.

### 3.3. UV–Vis Absorption Measurements

UV–Vis spectra titrations were performed using 0.01 M Tris buffer, pH 7.6. Calf thymus DNA (ctDNA) was purchased from Sigma-Aldrich (Saint Louis, MO, USA) and used without further purification. The solution of ctDNA in Tris buffer was sonicated for 5 min and the DNA concentration was determined using the molar extinction coefficient 6600 M^−1^·cm^−1^ at 260 nm [52]. The DNA purity was determined by monitoring the value of the A_260_/A_280_ ratio. DNA concentration was expressed as micromolar equivalents of the base pairs. Acridine-thiosemicarbazone derivatives were dissolved in DMSO at a concentration of 1 mM (stock solution) from which working solutions of concentrations ranging from 10 to 50 μM were prepared by dilution using Tris buffer. After compound concentration optimization, ctDNA titration was performed with constant acridine derivative concentrations. All measurements were performed at 25 °C in a rectangular quartz cuvette with a 1 cm path length. The intrinsic binding constant (K_b_) was obtained by fitting the data to Equation (1) [40]:

[DNA]/(ε_a_ − ε_f_) = [DNA]/(ε_b_ − ε_f_) + 1/K_b_ (ε_b_ − ε_f_) (1)

where ε_a_, ε_b_ and ε_f_ are the apparent, bound with DNA, and free extinction coefficients of compounds, respectively. ε_a_, ε_b_ and ε_f_ are all calculated from the Lambert–Beer’s law (ε = A/[compound]). Plot fitting of [DNA]/(ε_a_ − ε_f_) *vs.* [DNA] used K_b_ obtained from the ratio of the slope to the *y* intercept. The binding data were fitted using the Origin 8.0^®^ software (OriginLab Corporation, Northampton, MA, USA).

### 3.4. Fluorescence Measurements

Fluorescence measurements of non-bound derivatives were performed with solution concentration of 15 μM in 0.01 M Tris buffer, pH 7.6. Emission spectra were recorded in the region 380–600 nm using an excitation wavelength of 356–364 nm. All measurements were performed at 25 °C in a rectangular quartz cuvette with a 1 cm path length. Fluorescence intensities were expressed in arbitrary units. Fluorescence titrations were conducted by the addition of increasing amounts of ctDNA (0–120 μM bp) directly into the cell containing derivative solutions. Fluorescence intensities of compound solutions exposed to different ctDNA concentrations were used to calculate the Stern–Volmer constant, K_SV_, by the following Equation (2) [46]:

F_0_/F = 1 + K_SV_[Q] (2)

where F_0_ and F are the steady-state fluorescence intensities of compounds in the absence and in the presence of quencher (ctDNA), K_SV_ is the Stern–Volmer quenching constant, and [Q] is the concentration of quencher.

### 3.5. Determination of Antiproliferative Activity

Nine human cancer cell lines (U251 (glioma, CNS), MCF-7 (breast), NCI-ADR/RES (ovarian expressing phenotype multiple drugs resistance), 786-O (kidney), NCI-H460 (lung, non-small cells), PC-3 (prostate), OVCAR-03 (ovarian), HT-29 (colon adenocarcinoma) and K-562 (chronic myeloid leukemia)) and a normal cell line HaCat (human keratinocyte) were kindly provided by Frederick Cancer Research & Development Center, National Cancer Institute, Frederick, MA, USA. Stock cultures were grown in 5 mL of RPMI-1640 (GIBCO, Carlsbad, CA, USA) supplemented with 5% fetal bovine serum (FBS, GIBCO BRL). Penicillin: streptomycin mixture 1000 U/mL:1000 µg/mL (1 mL/L RPMI-1640) was added to experimental cultures. Cells in 96-well plates (100 µL cells/well, cell densities can be seen in Table S1) were exposed to different concentrations of samples (0.25, 2.5, 25 and 250 µg/mL) in DMSO/RPMI/FBS 5% at 37 °C, 5% CO_2_, for 48 h. Final DMSO concentration did not affect cell viability. Cells were then fixed with trichloroacetic acid solution (50%, *v*/*v*) and cell proliferation was determined by spectrophotometric quantification (540 nm) of cellular protein content using sulforhodamine B assay [49]. Doxorubicin (DOX; 0.025–25 µg/mL) and *m*-AMSA at the same concentration were used as a positive control. Three measurements were obtained: at the beginning of incubation (T_0_) and 48 h post-incubation for compound-free (C) and tested (T) cells. The choice of 48 h incubation was based on the NCI60 protocol, proposed by NCI/EUA for antiproliferative screening. Cell proliferation was determined according to the equation:

Cell proliferation = 100 × [(T − T_0_)/C − T_0_] (3)

Cytostatic effect was observed when T_0_ ≤ T < C while cytocidal effect occurred when T < T_0_. From the concentration–response curve for each cell line, GI_50_, TGI, and LC_50_ values were determined through nonlinear regression analysis using the software Origin 8.0 (OriginLab Corporation). The experiments were done in triplicate.

## 4. Conclusions

Eight new (*Z*)-2-(acridin-9-ylmethylene)-*N*-phenylhydrazinecarbothioamide derivatives (**3a**–**h**) proved binding constants with ctDNA in the range 1.74 × 10^4^ to 1.0 × 10^6^ M^−1^ and quenching constants from −0.2 × 10^4^ to 2.18 × 10^4^ M^−1^ indicating high affinity to ctDNA base pairs. Combinations of hyperchromic or hypochromic effect and red or blue shifts were observed for most compounds, except for **3g** derivative. The most efficient compound in binding to ctDNA *in vitro* was the chloro-substituted derivative **3f** which was demonstrated by hypochromism, red shift and fluorescence quenching. On the other hand, the non-substituted phenyl ring derivative **3a** showed the highest antiproliferative activity. In comparison with biochemical and biological properties (**3a**–**h)** produced in this study the following conclusions can be drawn: both binding constants with ctDNA and antiproliferative activities were influenced by substitution on the phenyl ring of thiosemicarbazone moieties since the most active compounds did not possess substitution (**3a**). There was no correlation between electron-withdrawing (**3c**, **3d** and **3e**) or electron-donating substituents on the phenyl ring (**3f**, **3g**) and antiproliferativy activity, since both dramatically decreased this property. The results indicate that the coupling of the acridine ring and the thiosemicarbazide moiety yielded new acridine-thiosemicarbazone derivatives with promising DNA binding. However, the characteristics of substituents on the phenyl ring influence both the DNA-binding and the antiproliferative properties.

## Supplementary Materials

Supplementary materials can be found at http://www.mdpi.com/1422-0067/16/06/13023/s1.
