# Supplementary material for: Synthesis, DNA Binding, and Antiproliferative Activity of Novel Acridine-Thiosemicarbazone Derivatives
Source: Int J Mol Sci. 2015 Jun 9;16(6):13023–42. doi: 10.3390/ijms160613023 (PMC4490484; doi:10.3390/ijms160613023)
Supplement: Supplementary file 1 [file ijms-16-13023-s001.pdf]

Supplementary Information

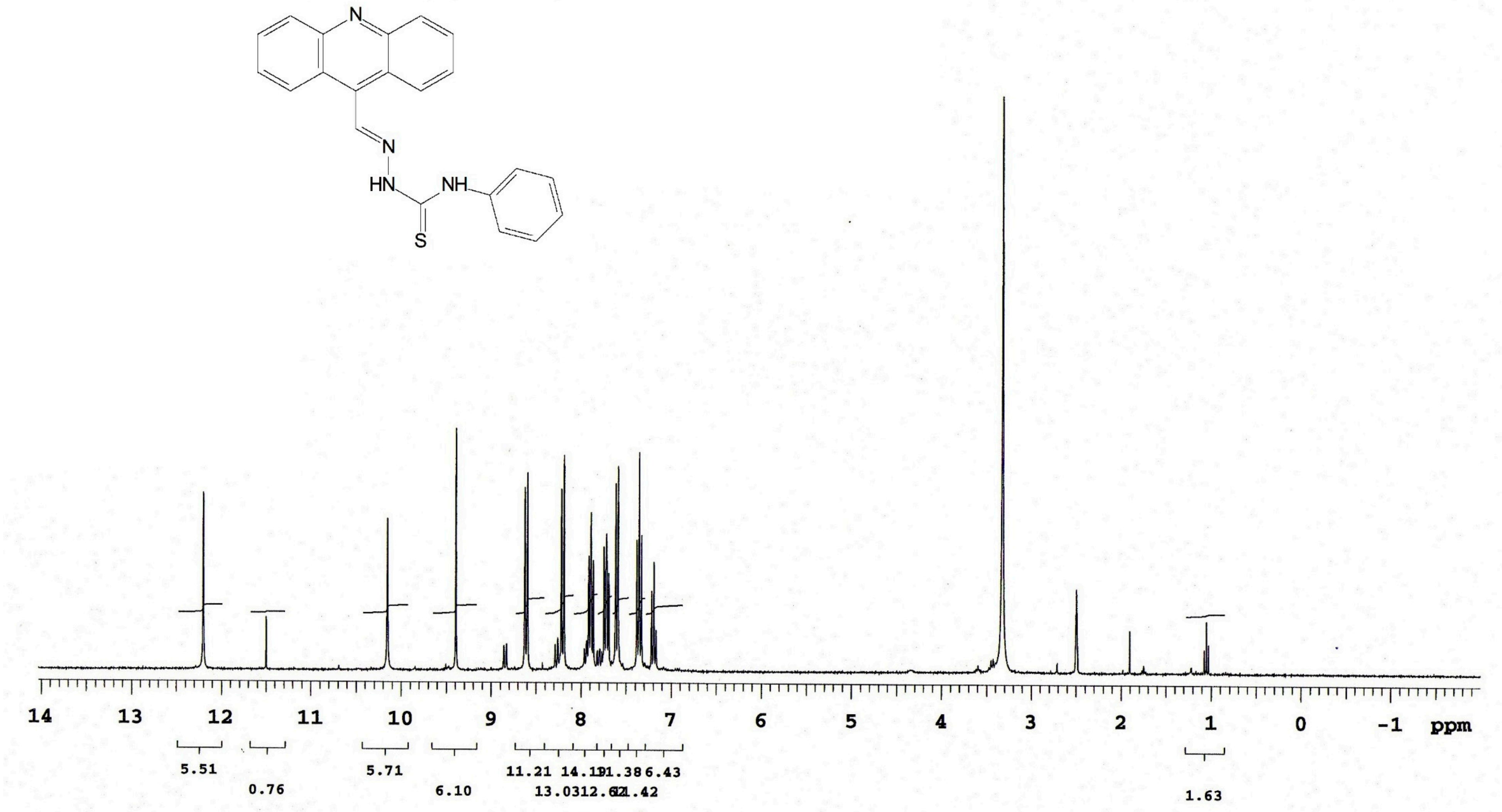

Figure S1. ^{1}H-NMR spectrum (DMSO) of derivative **3a**.

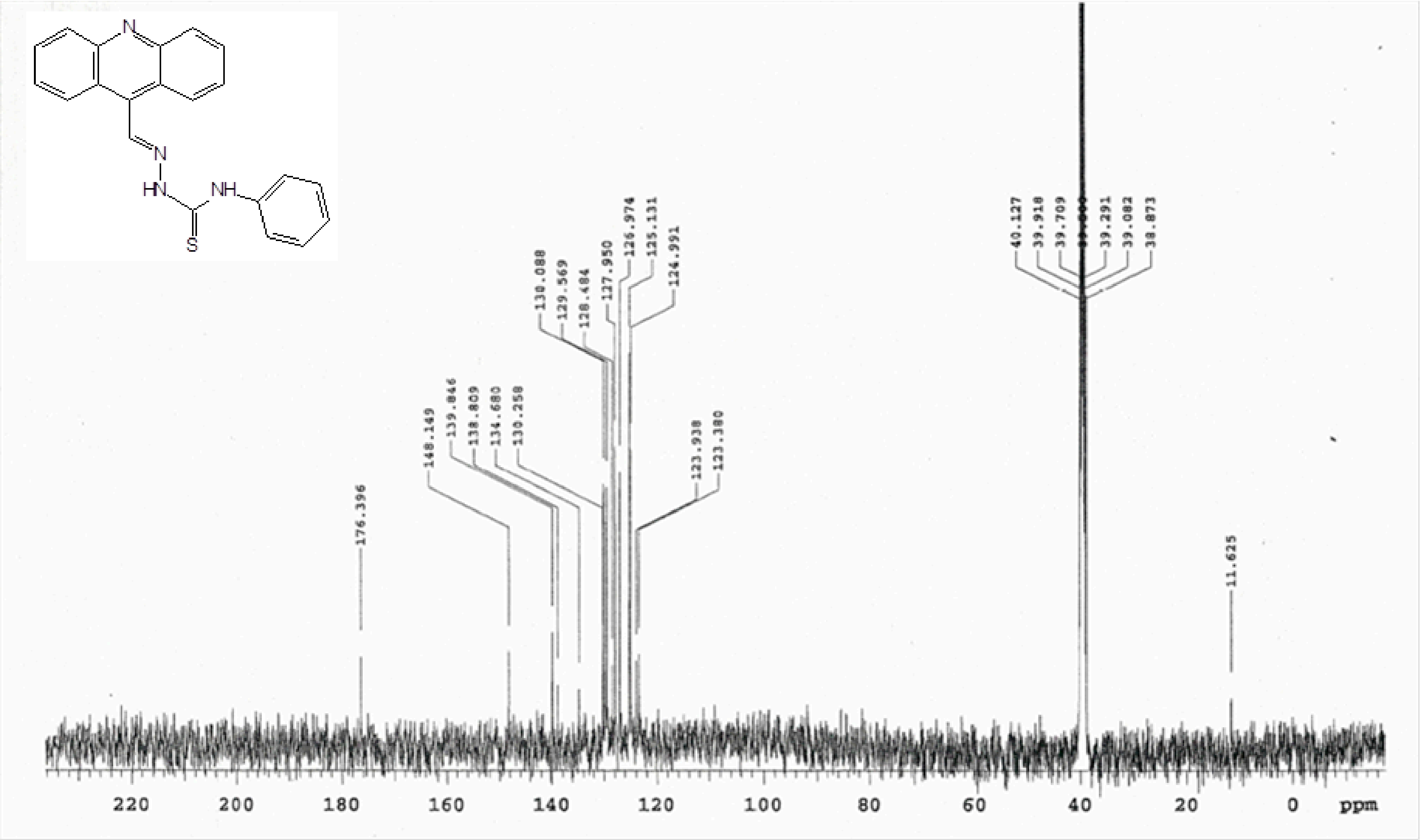

Figure S2. ^{13}C-NMR spectrum (DMSO) of derivative **3a**.

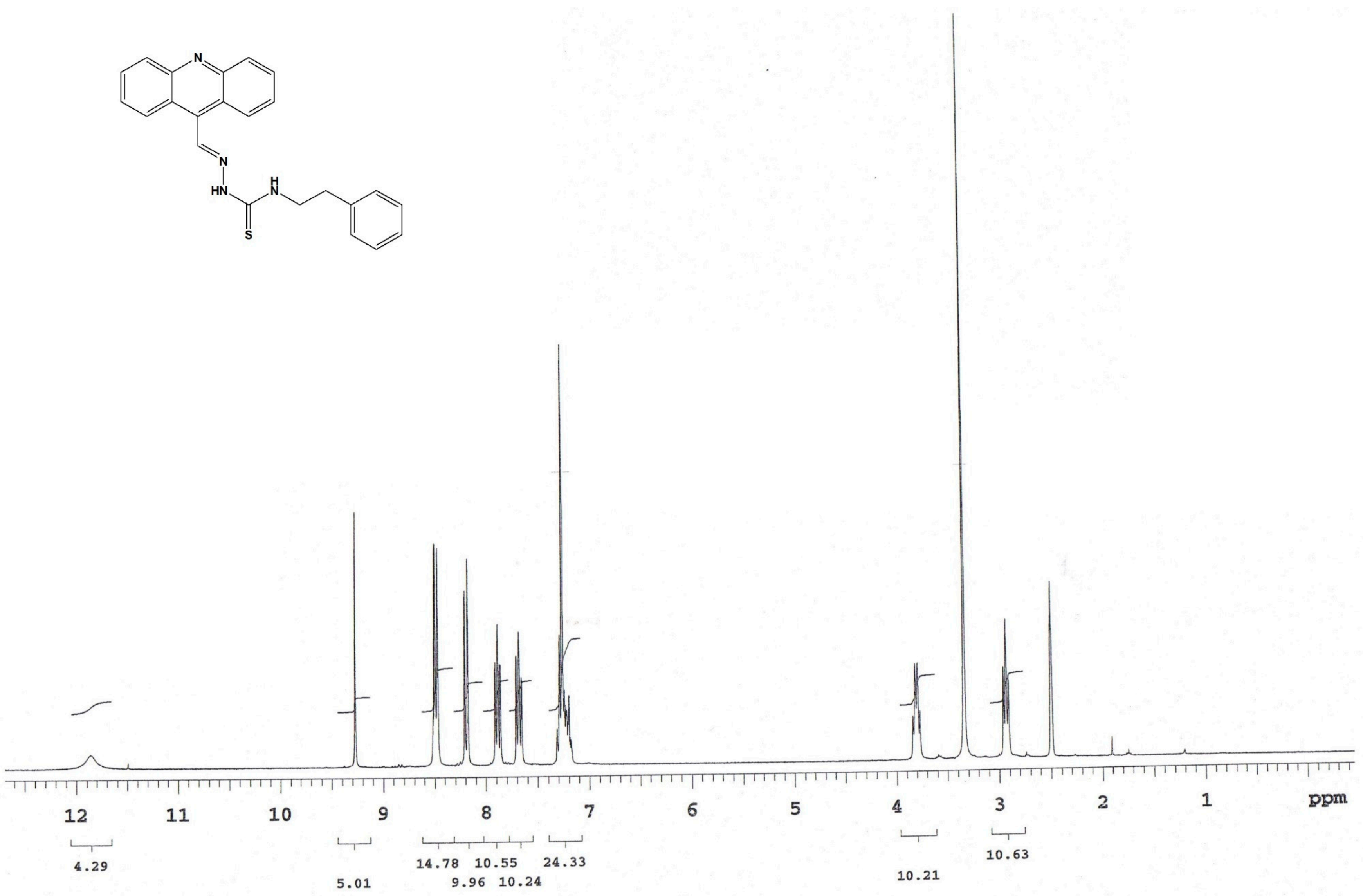

Figure S3. ^{1}H-NMR spectrum (DMSO) of derivative **3b**.

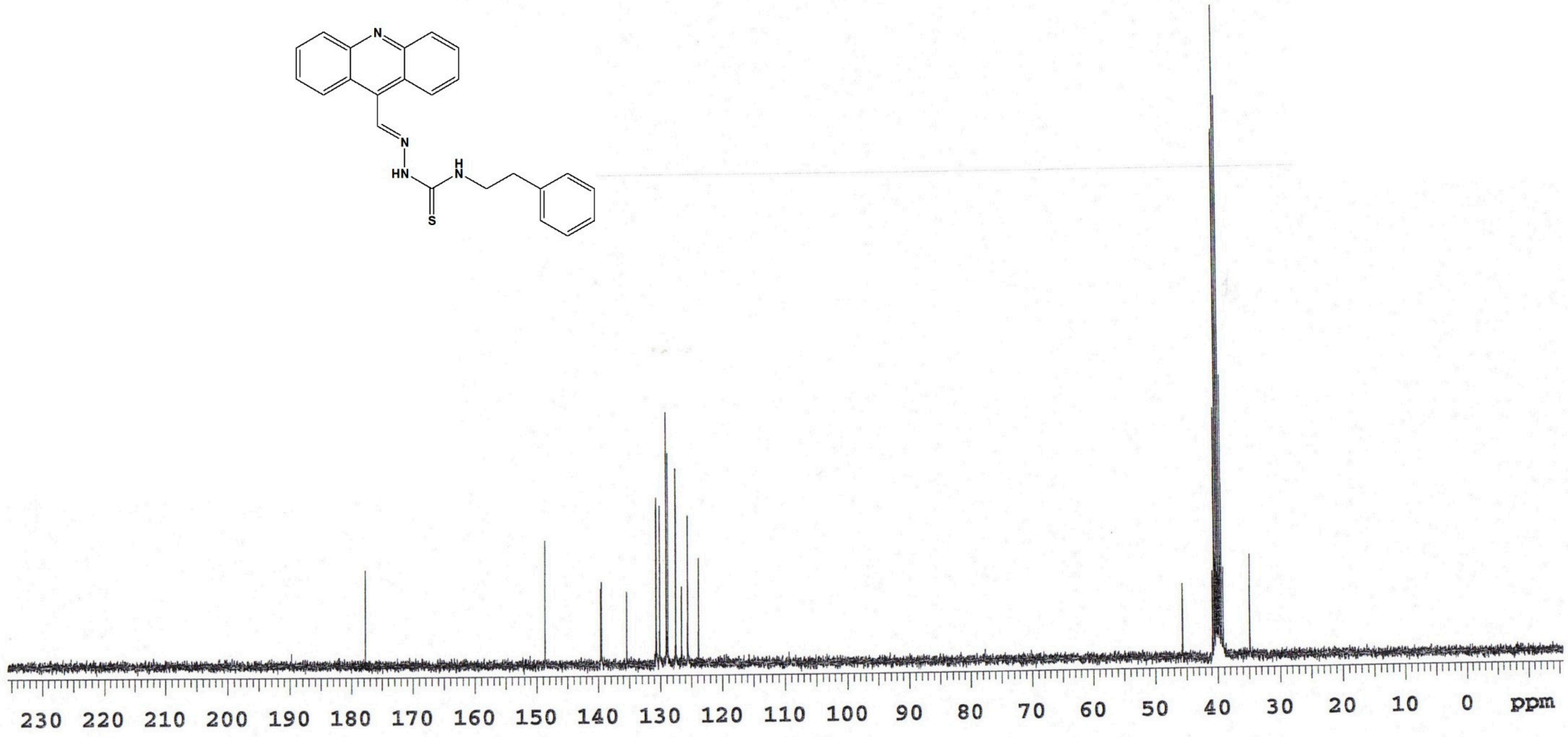

Figure S4. ^{13}C-NMR spectrum (DMSO) of derivative **3b**.

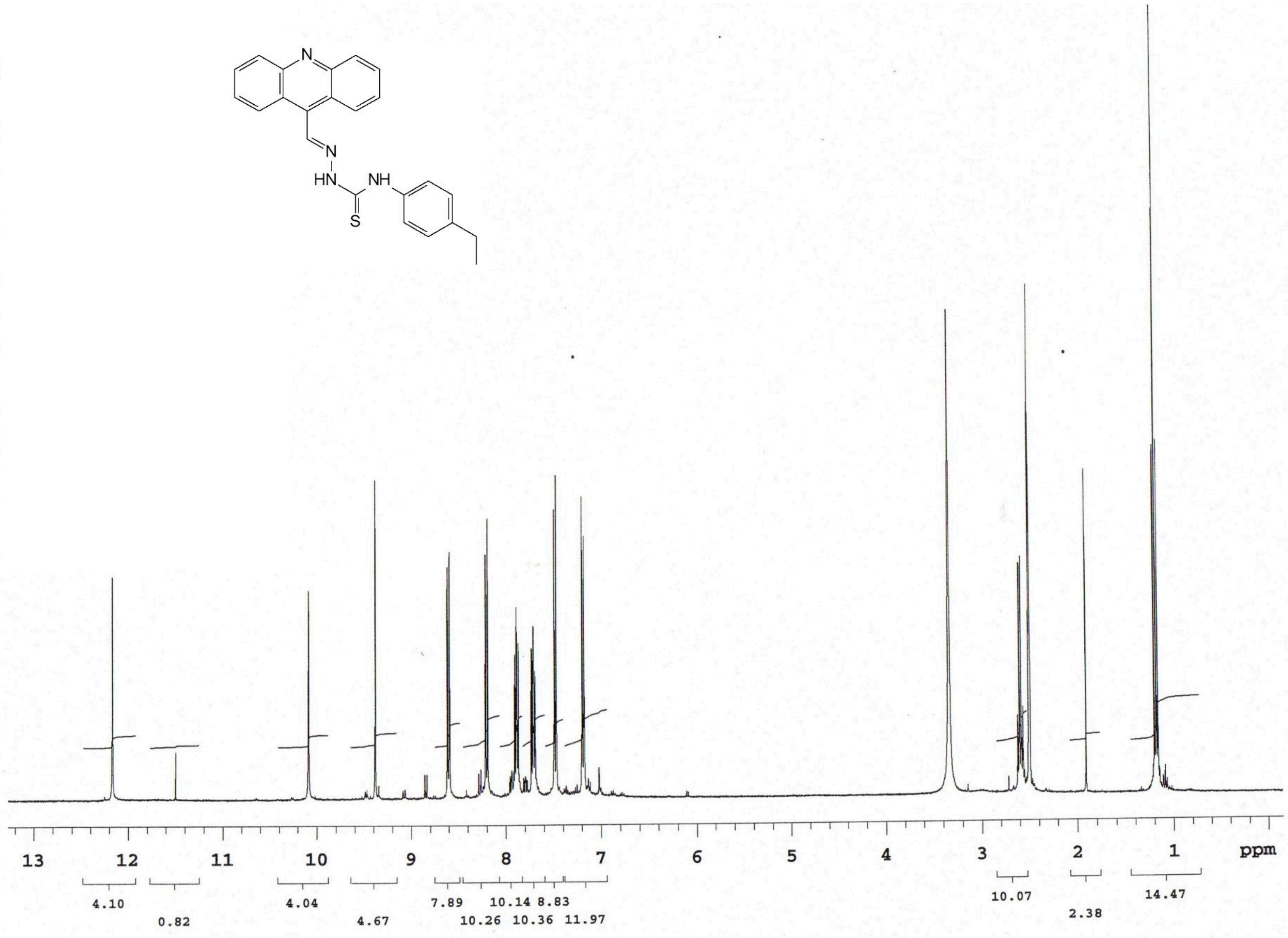

Figure S5. ^{1}H-NMR spectrum (DMSO) of derivative **3c**.

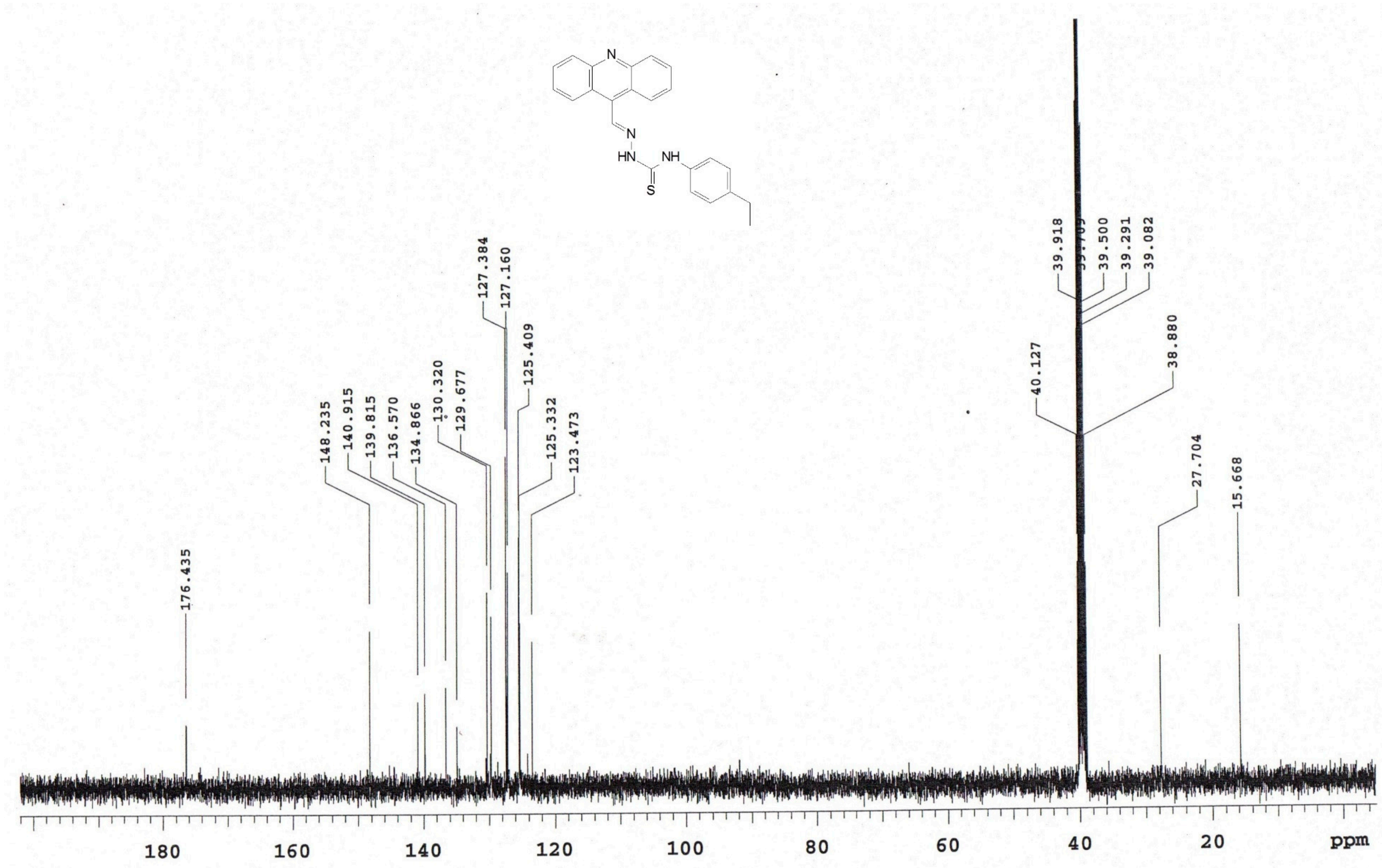

Figure S6. ^{13}C-NMR spectrum (DMSO) of derivative **3c**.

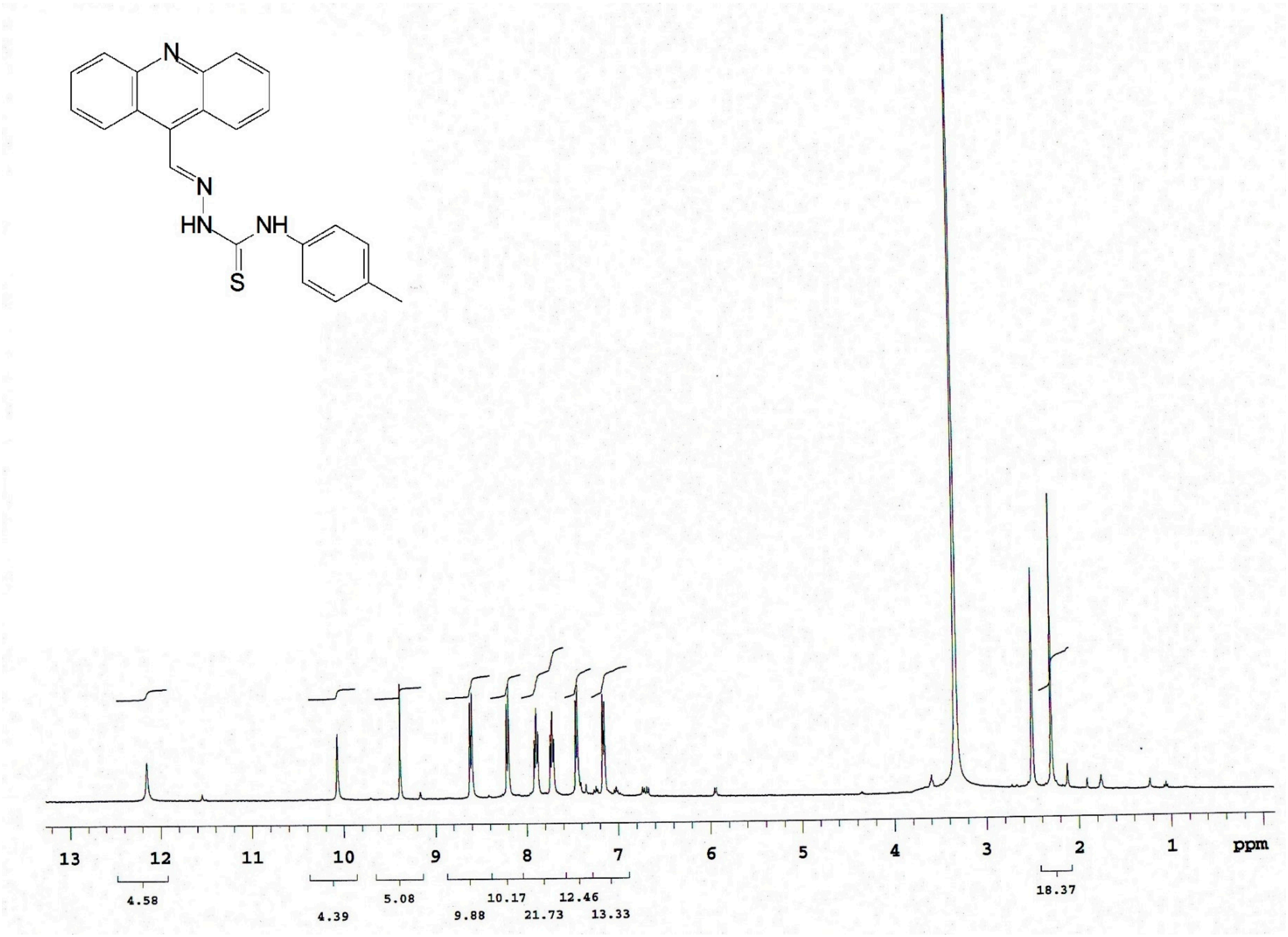

Figure S7. ^{1}H-NMR spectrum (DMSO) of derivative **3d**.

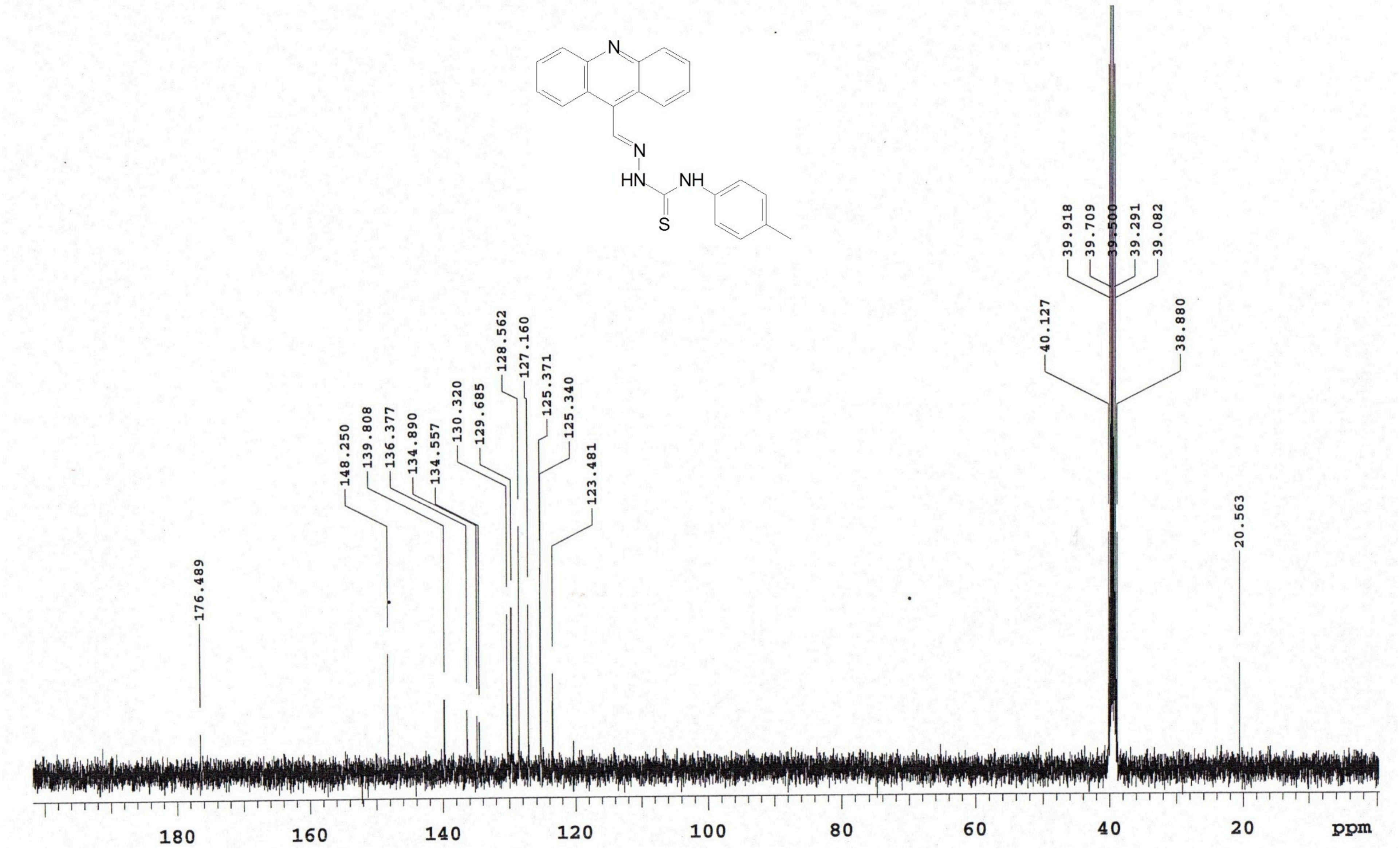

Figure S8. ^{13}C-NMR spectrum (DMSO) of derivative **3d**.

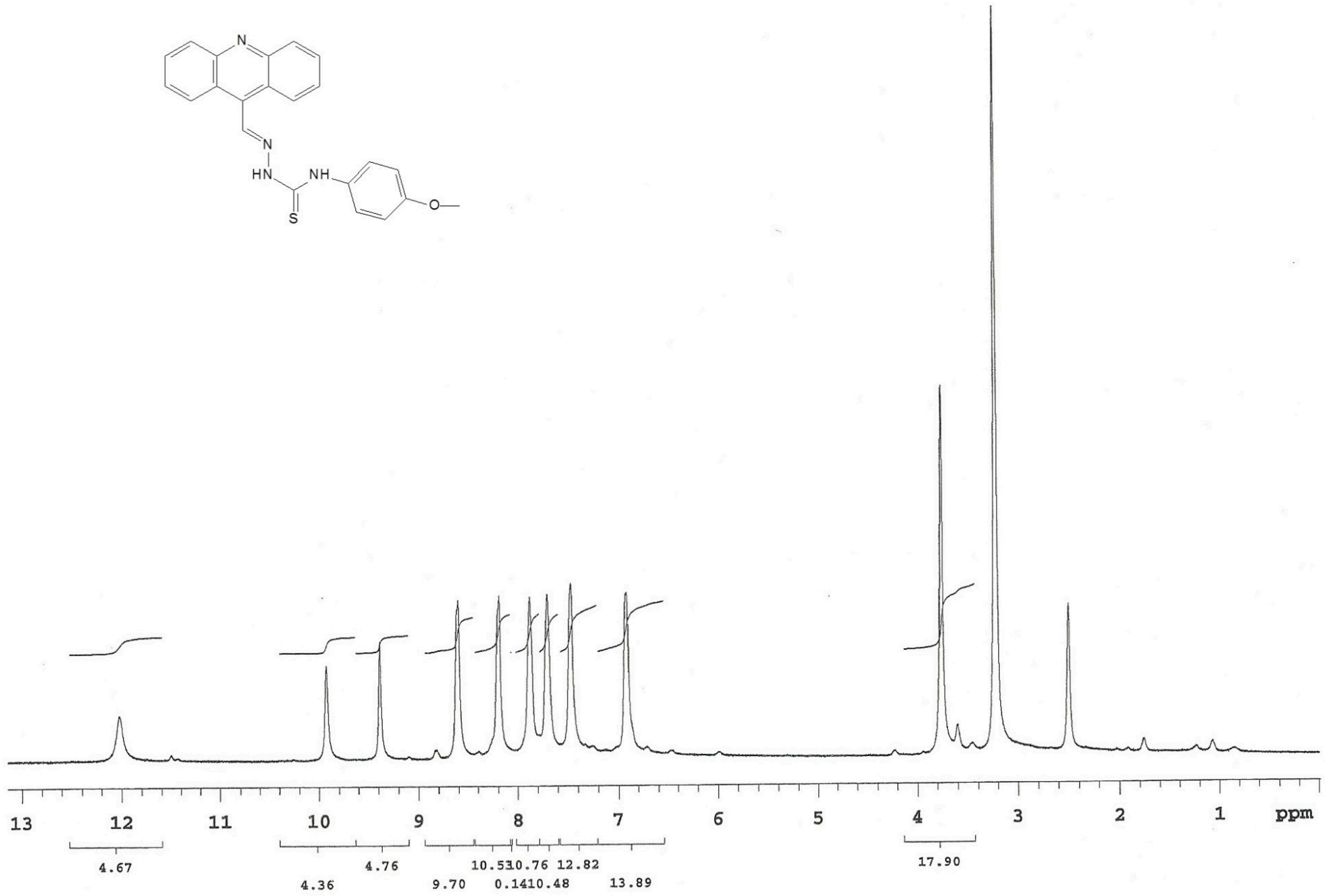

Figure S9. ^{1}H-NMR spectrum (DMSO) of derivative **3e**.

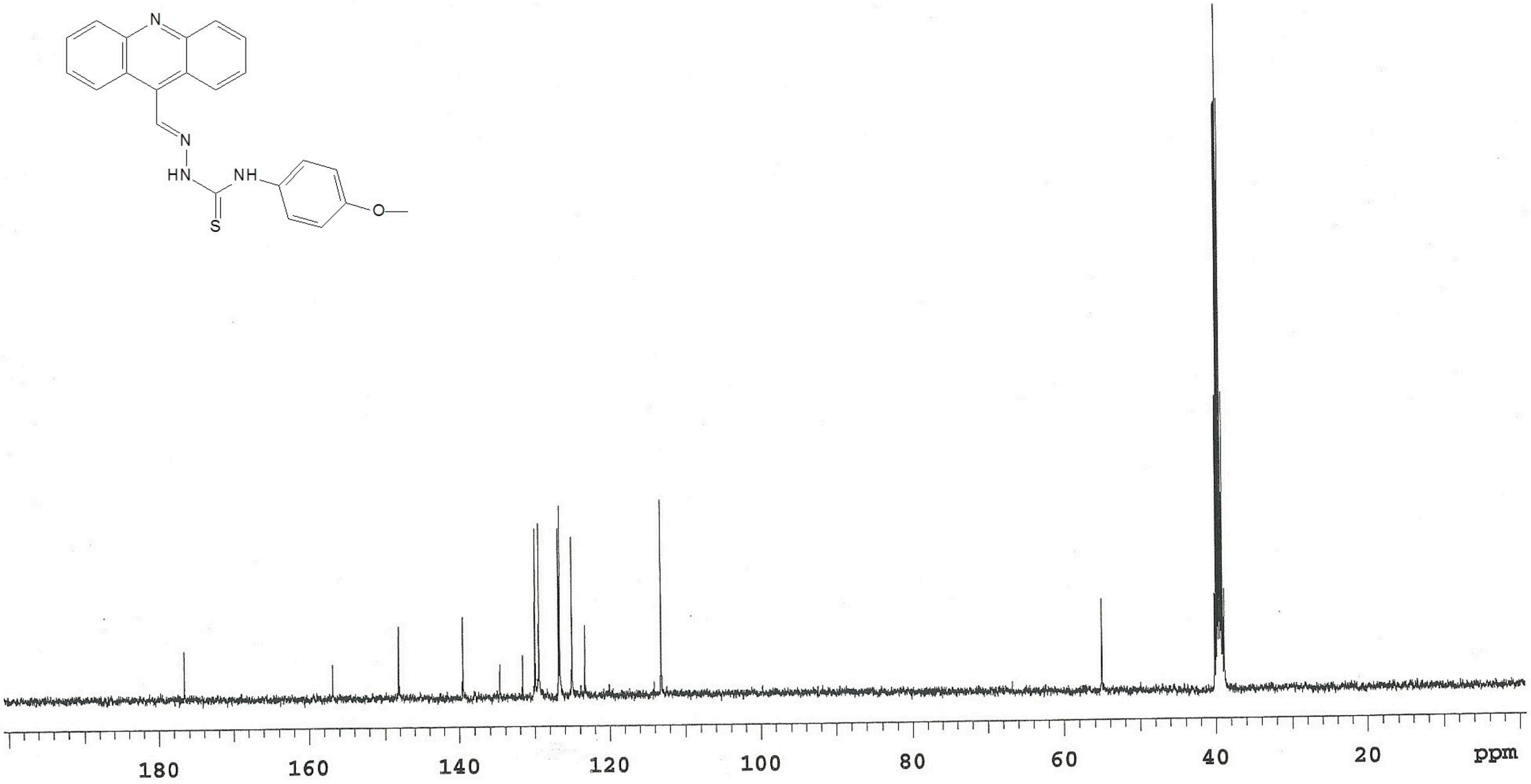

Figure S10. ^{13}C-NMR spectrum (DMSO) of derivative **3e**.

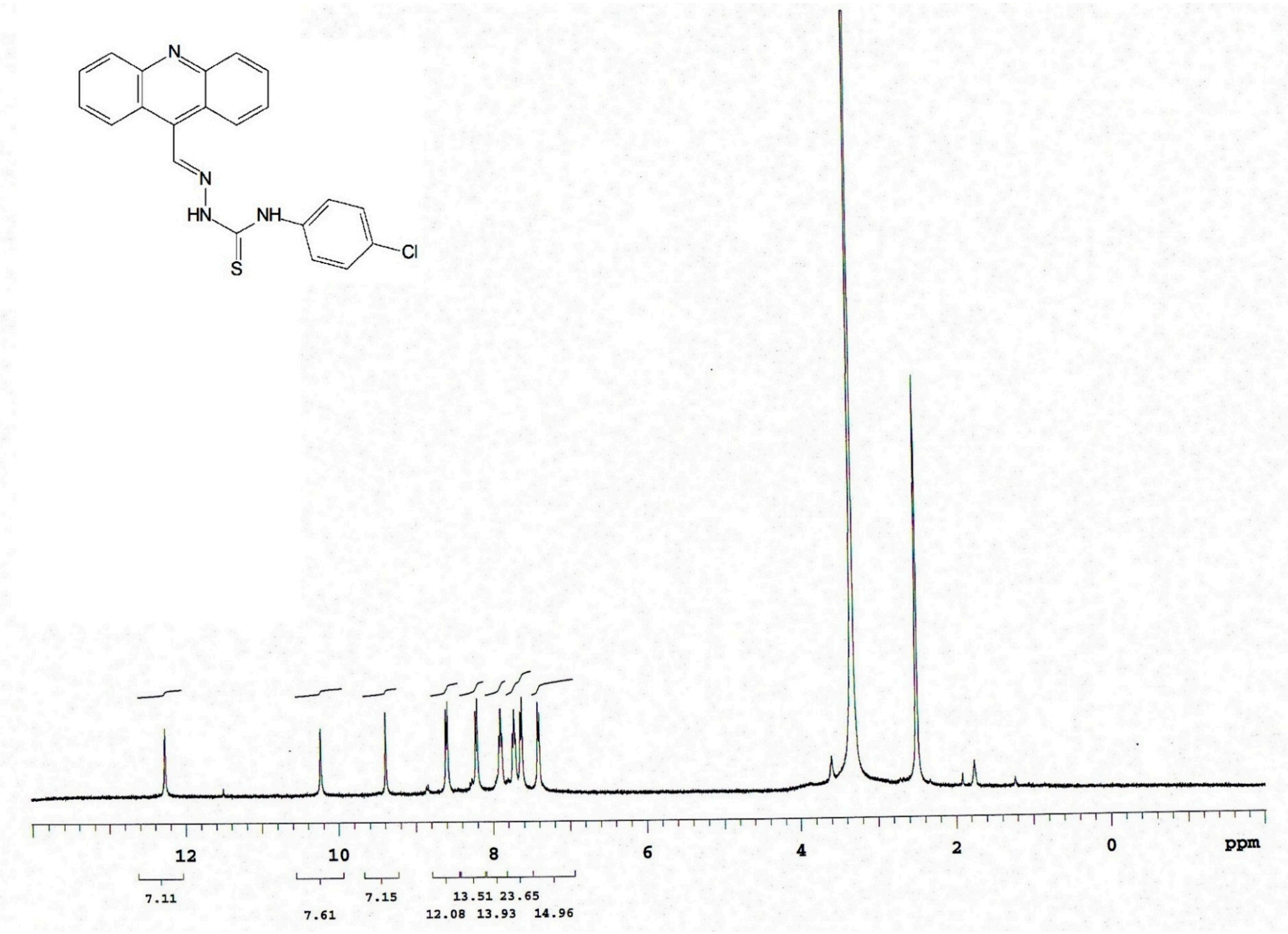

Figure S11. ^{1}H-NMR spectrum (DMSO) of derivative **3f**.

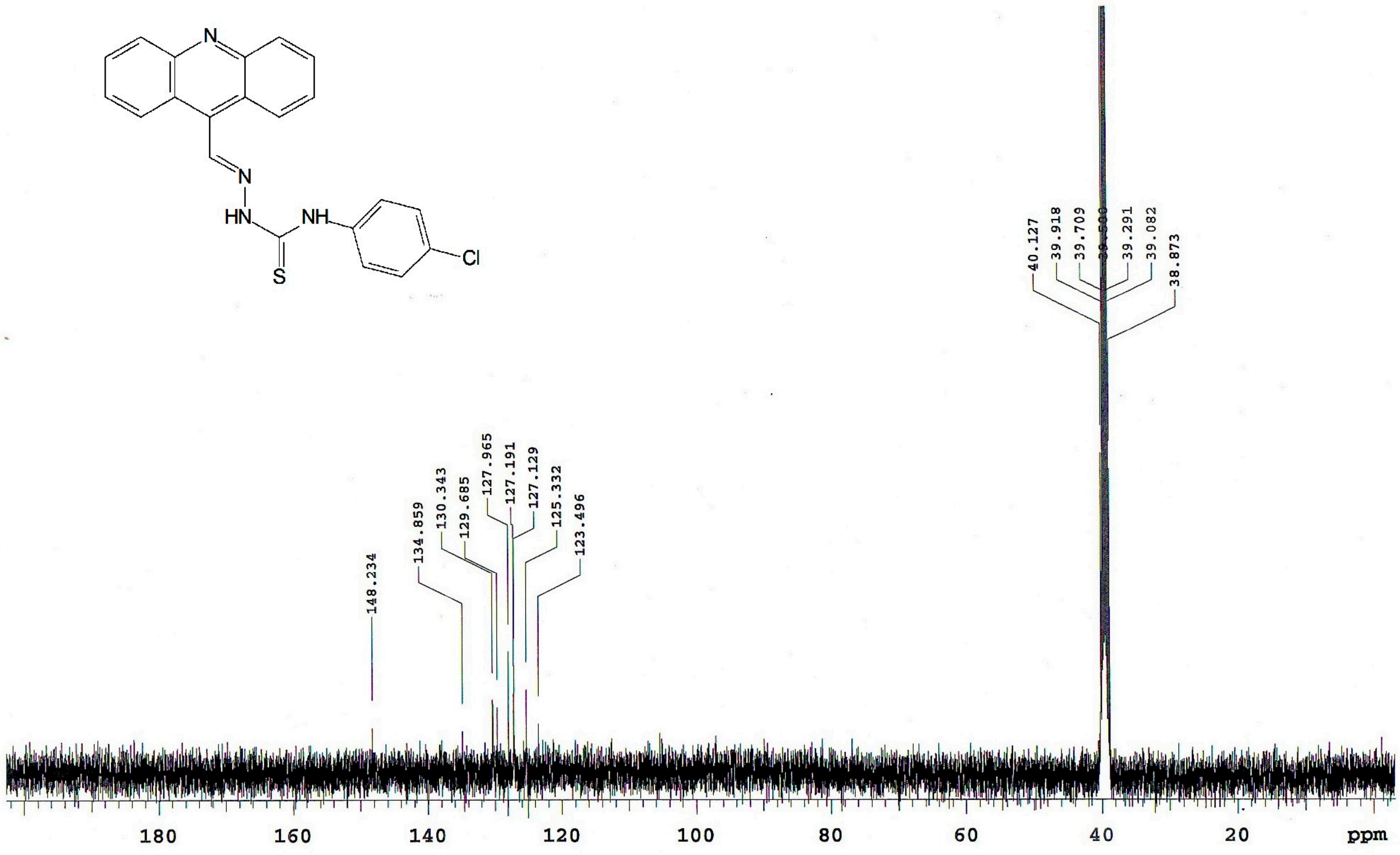

Figure S12. ^{13}C-NMR spectrum (DMSO) of derivative **3f**.

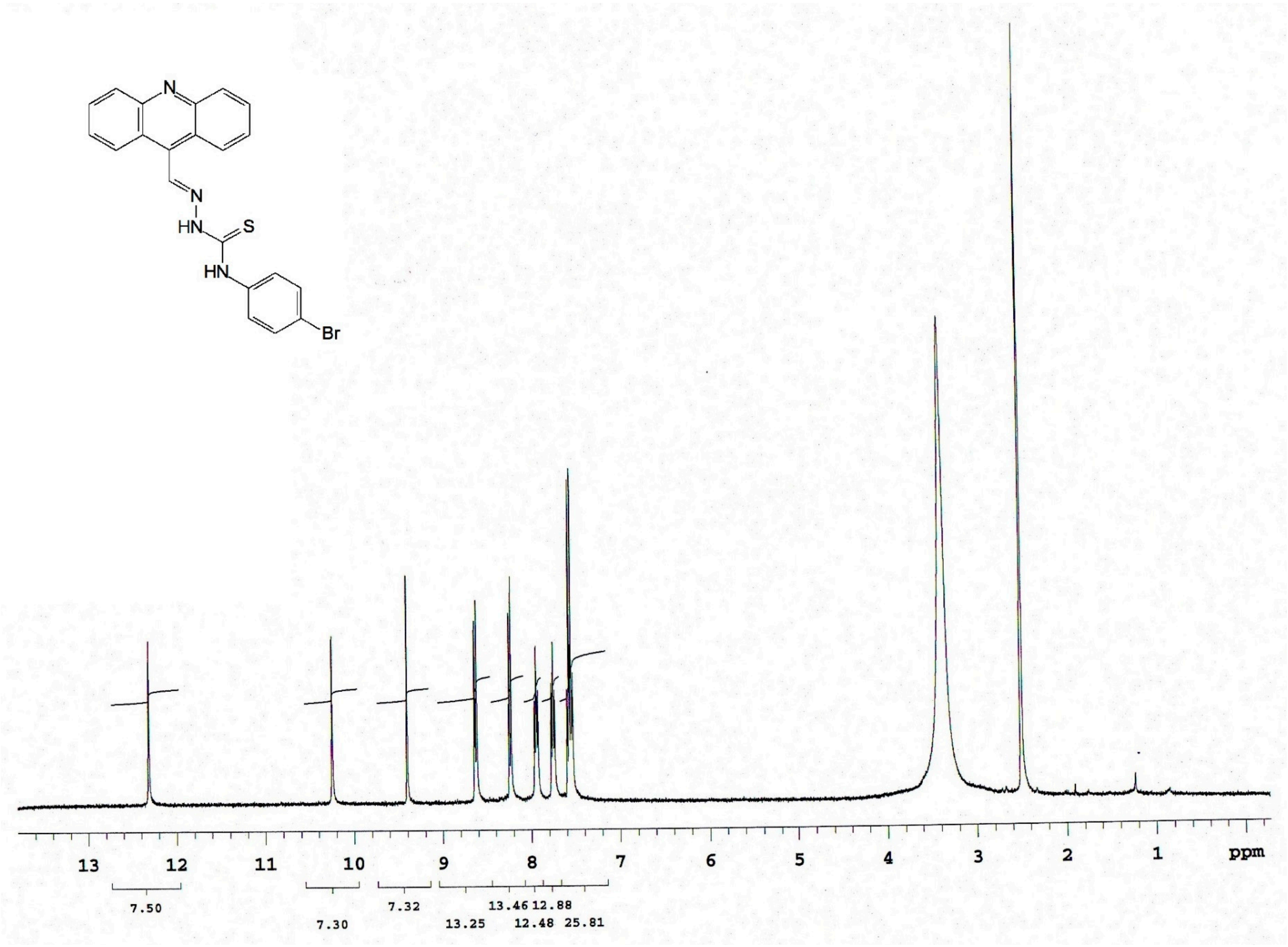

Figure S13. ^{1}H-NMR spectrum (DMSO) of derivative **3g**.

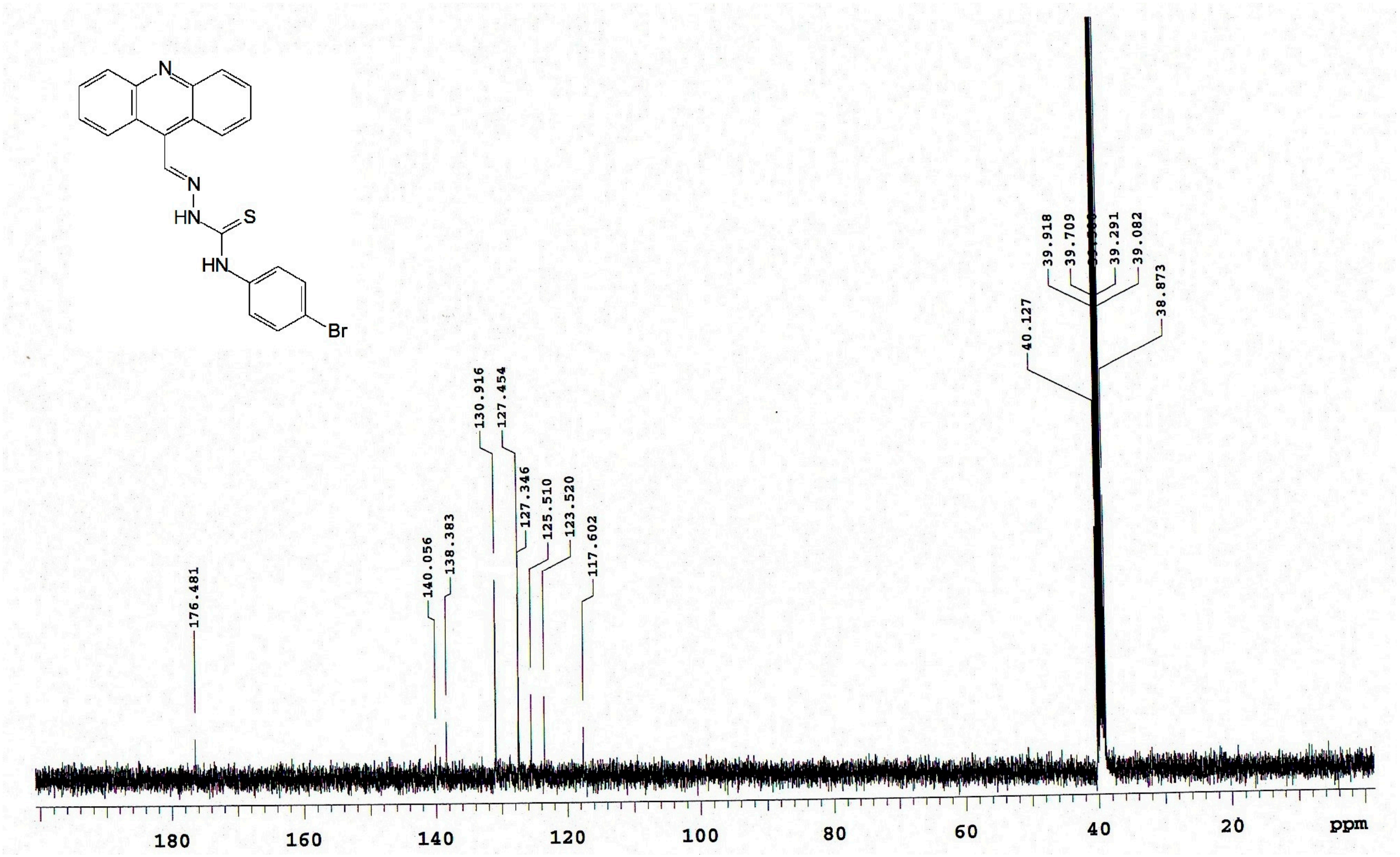

Figure S14. ^{13}C-NMR spectrum (DMSO) of derivative **3g**.

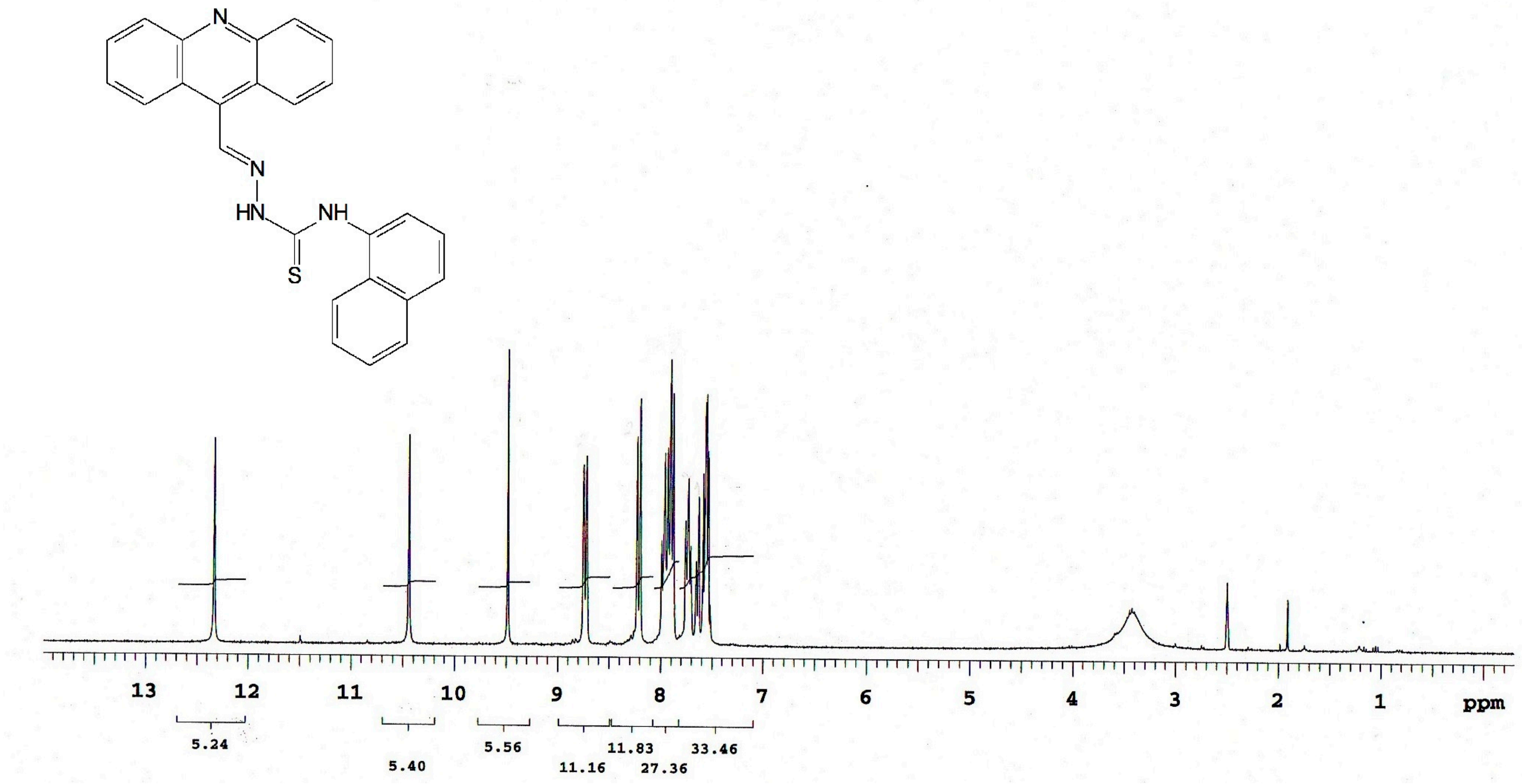

Figure S15. ^{1}H-NMR spectrum (DMSO) of derivative **3h**.

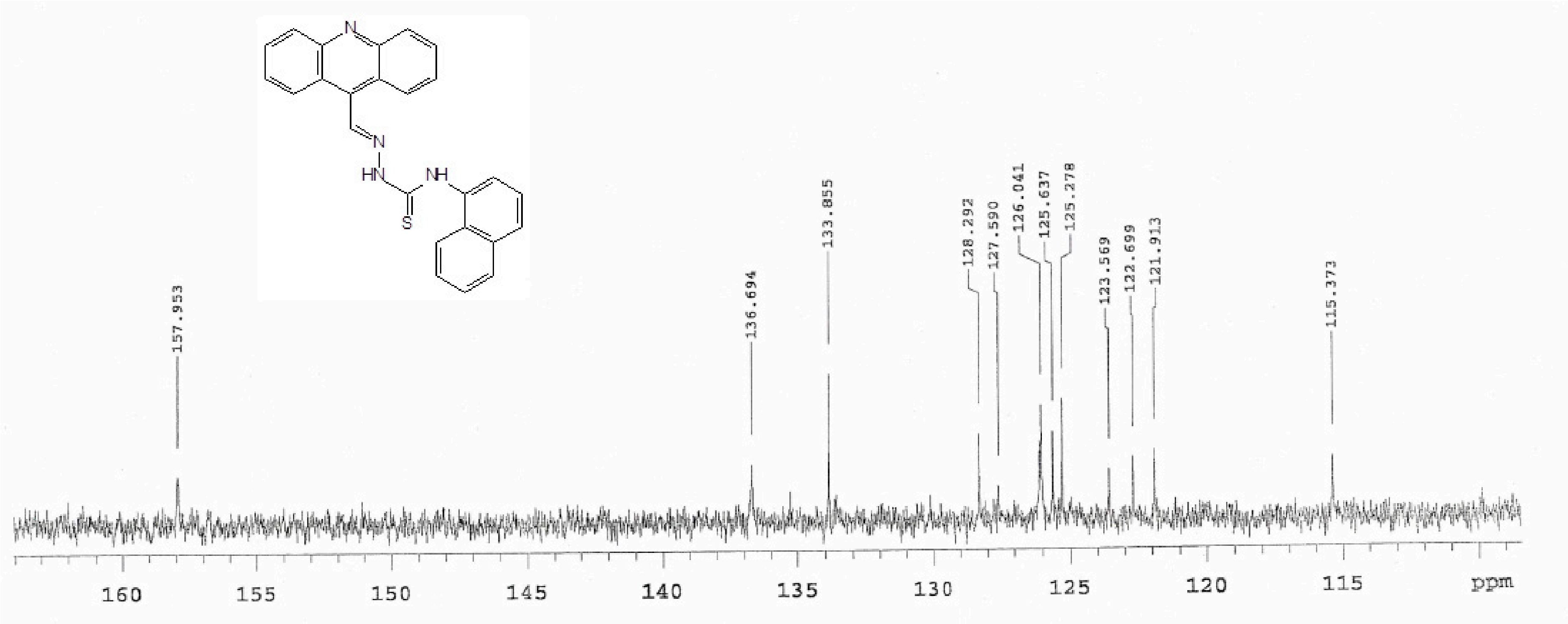

Figure S16. ^{13}C-NMR spectrum (DMSO) of derivative **3h**.

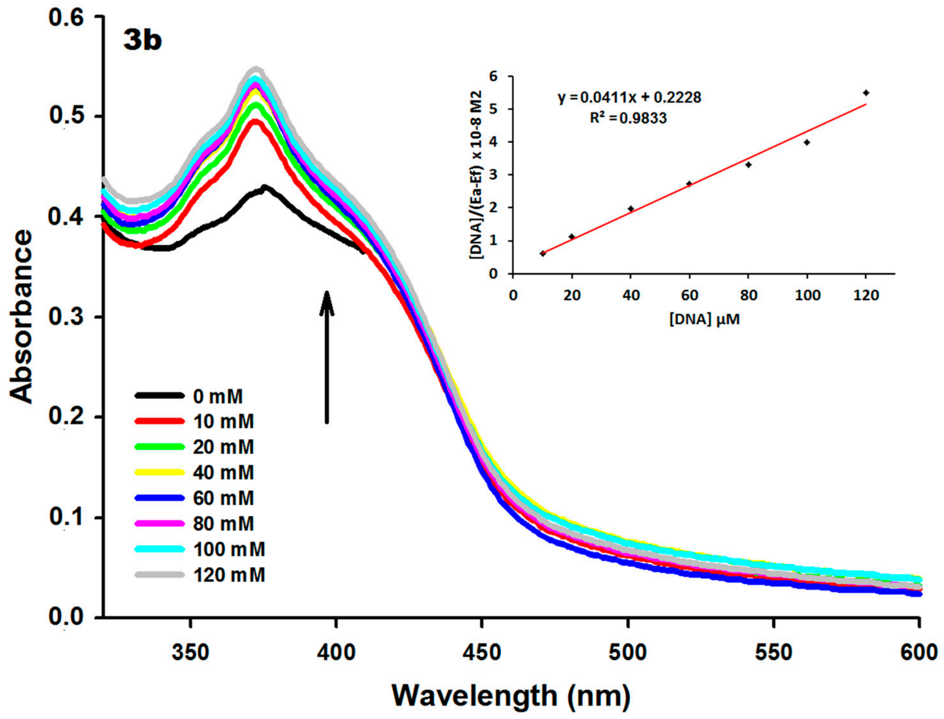

Figure S17. Absorption titration of derivative **3b** (50 µM) with increasing concentrations of ctDNA. [DNA] = 0, 10, 20, 40, 60, 80, 100 and 120 µM. Inset: corresponding to the plot of [DNA]/($\varepsilon_a - \varepsilon_f$) as function of DNA concentration as determined from the absorption spectral data. Arrow (↑) refers to hyperchromic effect.

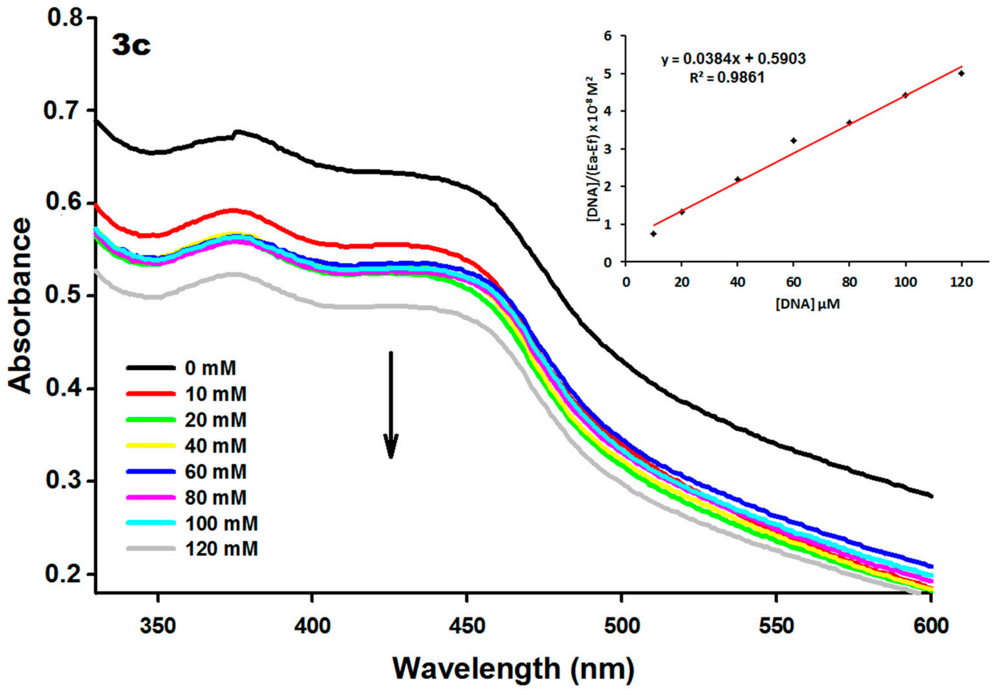

Figure S18. Absorption titration of derivative **3c** (50 µM) with increasing concentrations of ctDNA. [DNA] = 0, 10, 20, 40, 60, 80, 100 and 120 µM. Inset: corresponding to the plot of [DNA]/($\varepsilon_a - \varepsilon_f$) as function of DNA concentration as determined from the absorption spectral data. Arrow (↓) refers to hypochromic effect.

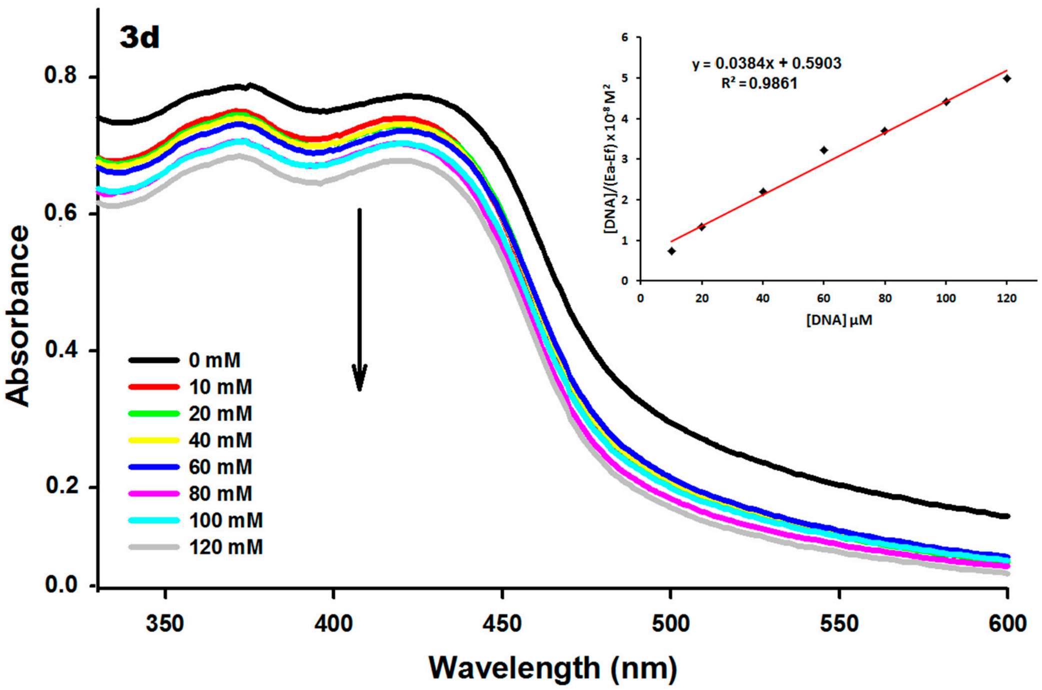

Figure S19. Absorption titration of derivative **3d** (50 µM) with increasing concentrations of ctDNA. [DNA] = 0, 10, 20, 40, 60, 80, 100 and 120 µM. Inset: corresponding to the plot of [DNA]/($\varepsilon_a - \varepsilon_f$) as function of DNA concentration as determined from the absorption spectral data. Arrow (↓) refers to hypochromic effect.

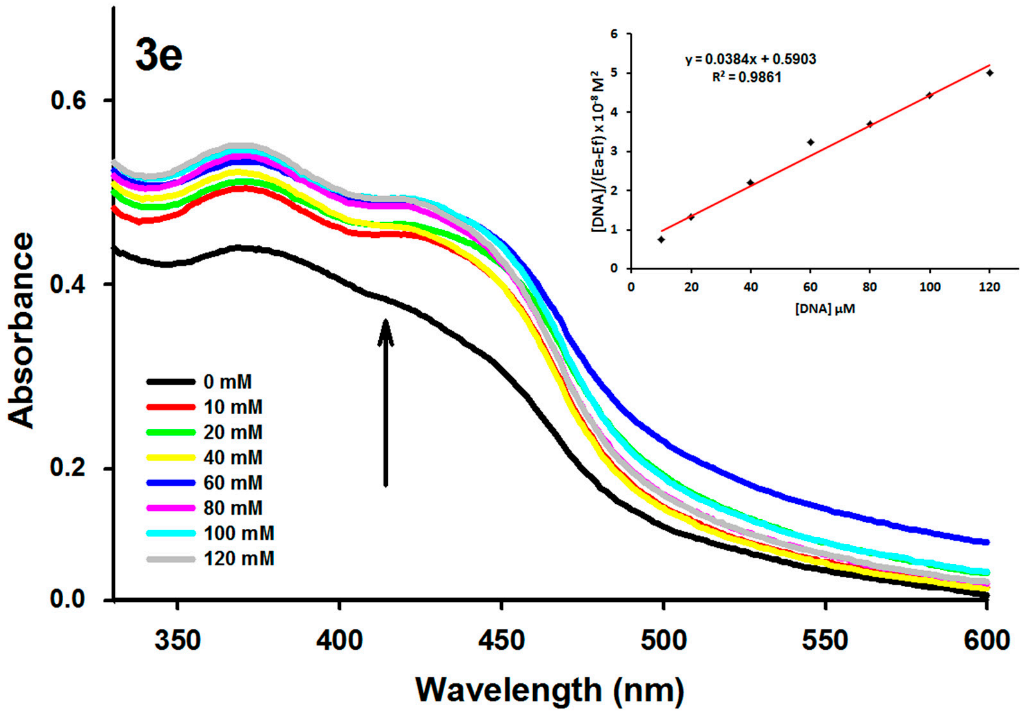

Figure S20. Absorption titration of derivative **3e** (50 µM) with increasing concentrations of ctDNA. [DNA] = 0, 10, 20, 40, 60, 80, 100 and 120 µM. Inset: corresponding to the plot of [DNA]/($\varepsilon_a - \varepsilon_f$) as function of DNA concentration as determined from the absorption spectral data. Arrow (↑) refers to hyperchromic effect.

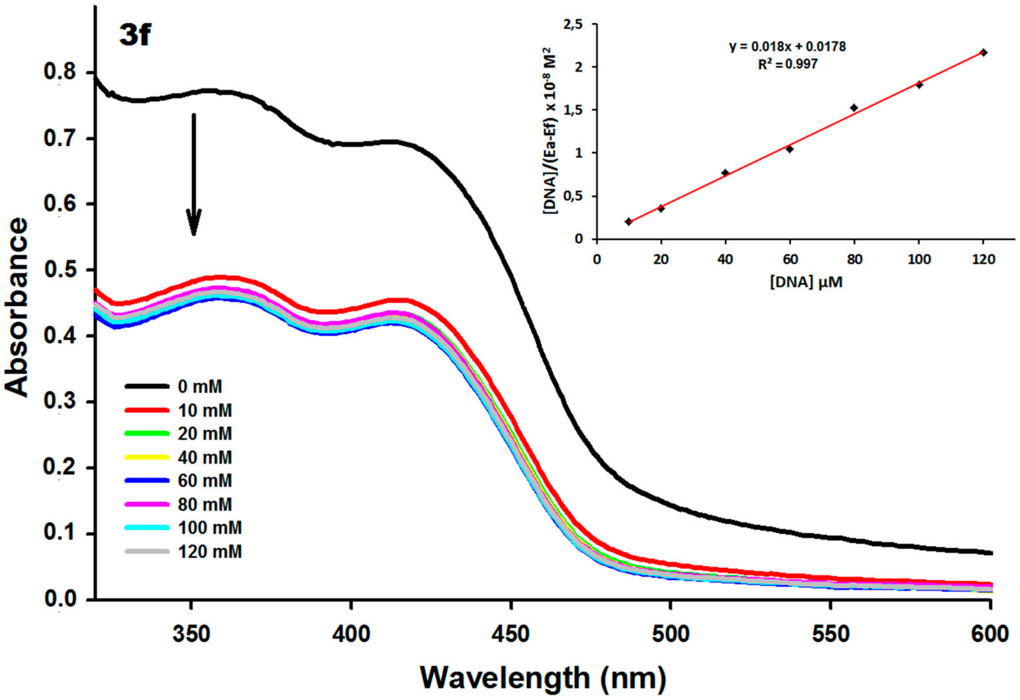

Figure S21. Absorption titration of derivative **3f** (50 μM) with increasing concentrations of ctDNA. [DNA] = 0, 10, 20, 40, 60, 80, 100 and 120 μM. Inset: corresponding to the plot of [DNA]/($\varepsilon_a - \varepsilon_f$) as function of DNA concentration as determined from the absorption spectral data. Arrow (↓) refers to hypochromic effect.

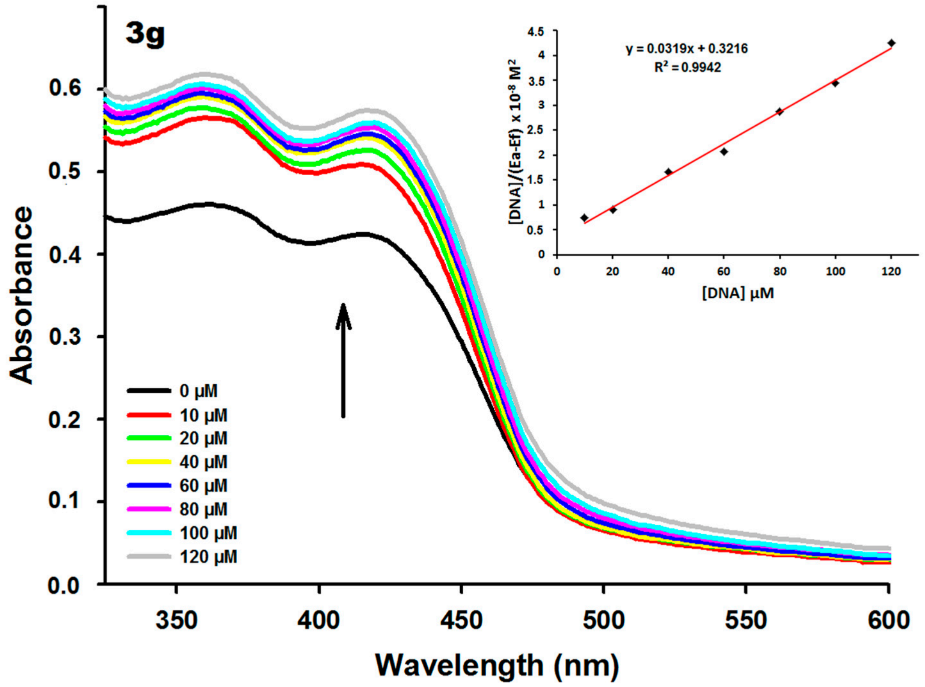

Figure S22. Absorption titration of derivative **3g** (50 μM) with increasing concentrations of ctDNA. [DNA] = 0, 10, 20, 40, 60, 80, 100 and 120 μM. Inset: corresponding to the plot of [DNA]/($\varepsilon_a - \varepsilon_f$) as function of DNA concentration as determined from the absorption spectral data. Arrow (↑) refers to hyperchromic effect.

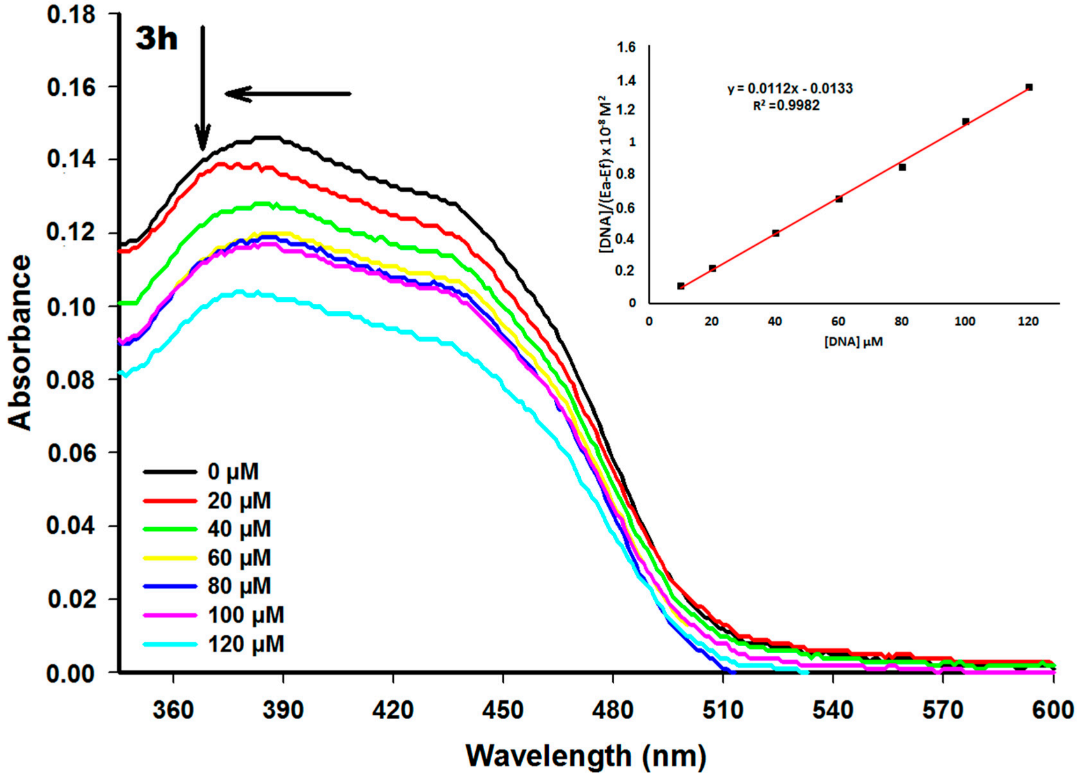

Figure S23. Absorption titration of derivative **3h** (50 µM) with increasing concentrations of ctDNA. [DNA] = 0, 10, 20, 40, 60, 80, 100 and 120 µM. Inset: corresponding to the plot of [DNA]/($\varepsilon_a - \varepsilon_f$) as function of DNA concentration as determined from the absorption spectral data. Arrows (↓) and (⟵) refer to hypochromic, and hypsochromic effects, respectively.

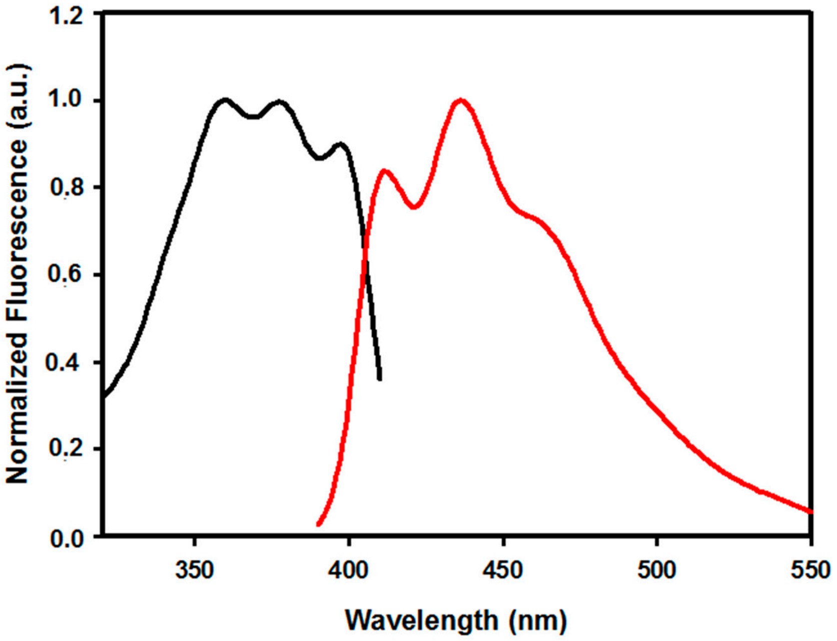

Figure S24. Excitation (black) and emission (red) spectra of derivative **3a** at concentrations of 15 µM in Tris-HCl buffer. Excitation at 359 nm and emission at 439 nm.

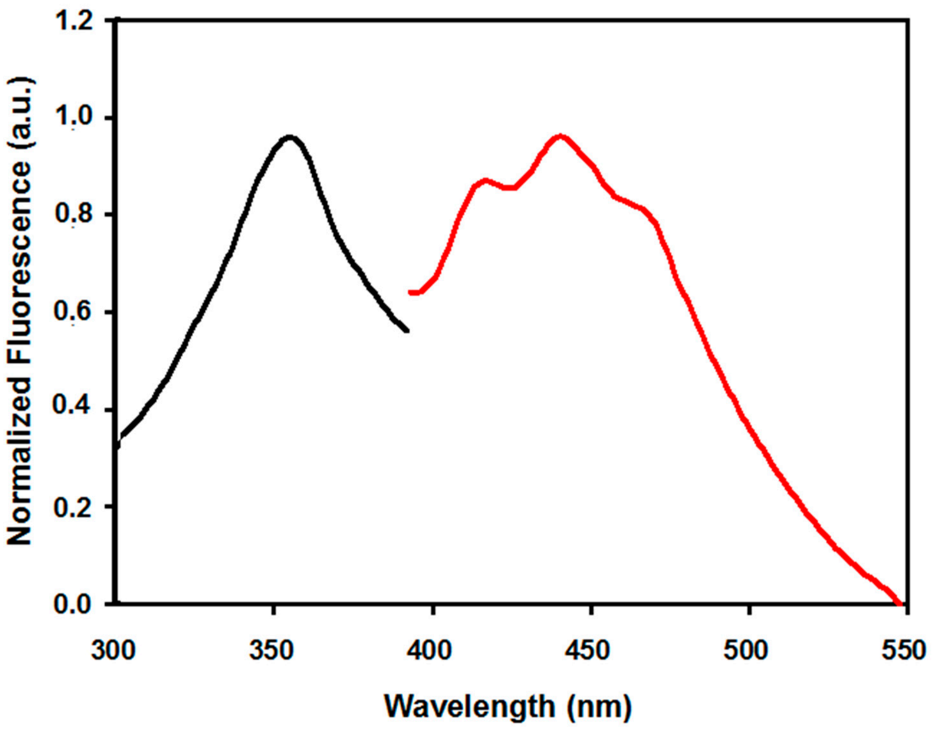

Figure S25. Excitation (black) and emission (red) spectra of derivative **3b** at concentrations of 15 µM in Tris-HCl buffer. Excitation at 370 nm and emission at 441 nm.

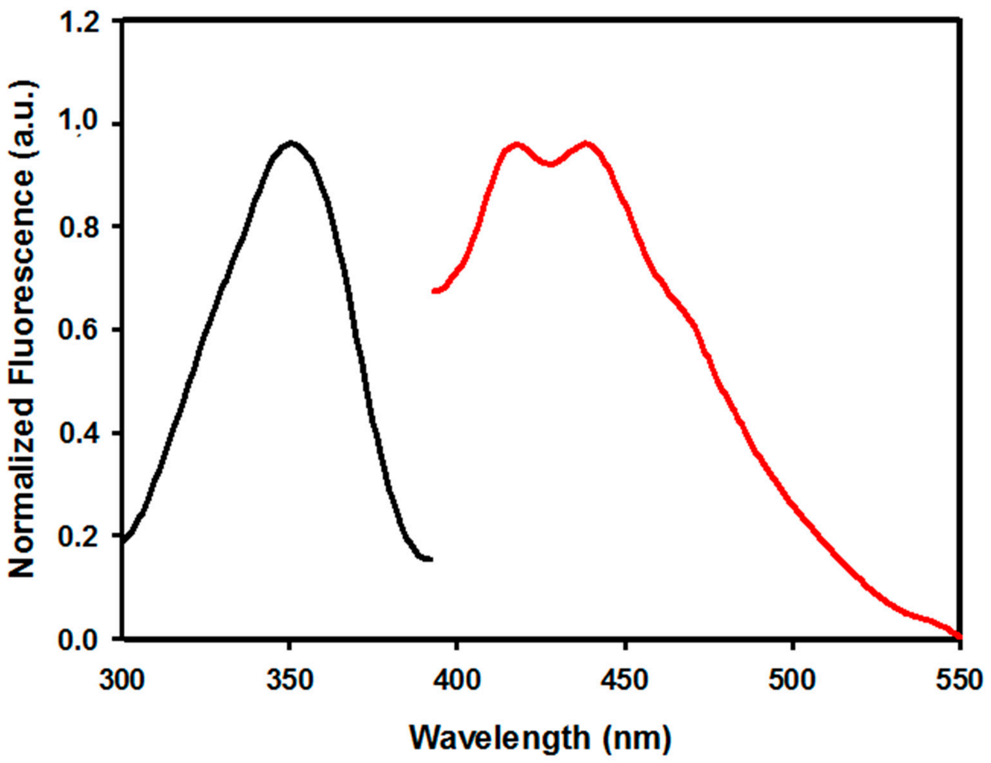

Figure S26. Excitation (black) and emission (red) spectra of derivative **3c** at concentrations of 15 µM in Tris-HCl buffer. Excitation at 370 nm and emission at 440 nm.

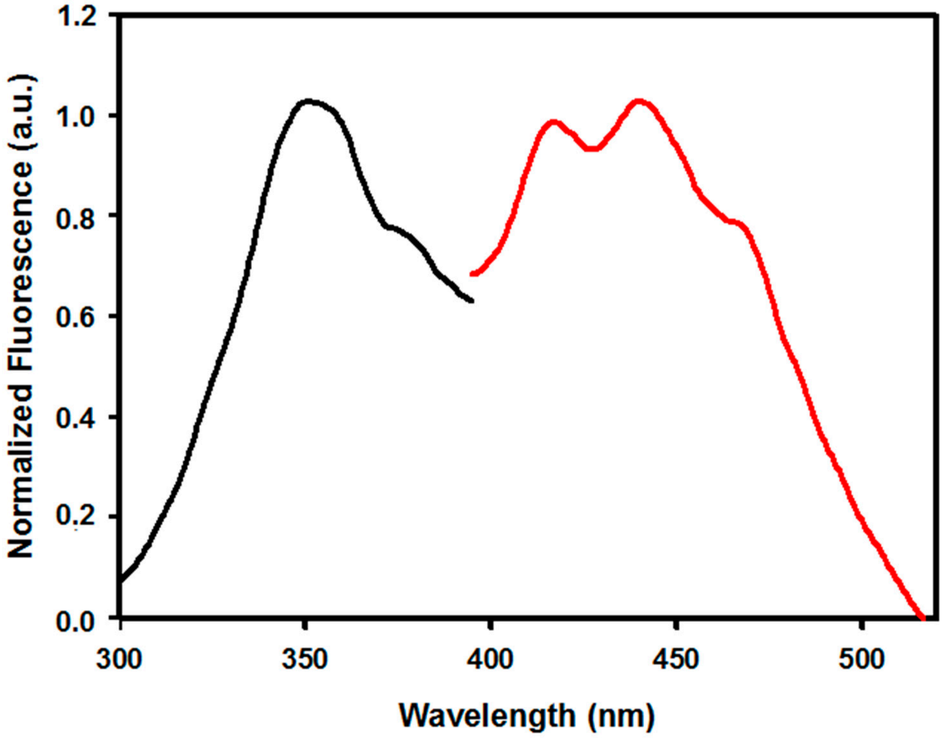

Figure S27. Excitation (black) and emission (red) spectra of derivative **3d** at concentrations of 15 μM in Tris-HCl buffer. Excitation at 370 nm and emission at 441 nm.

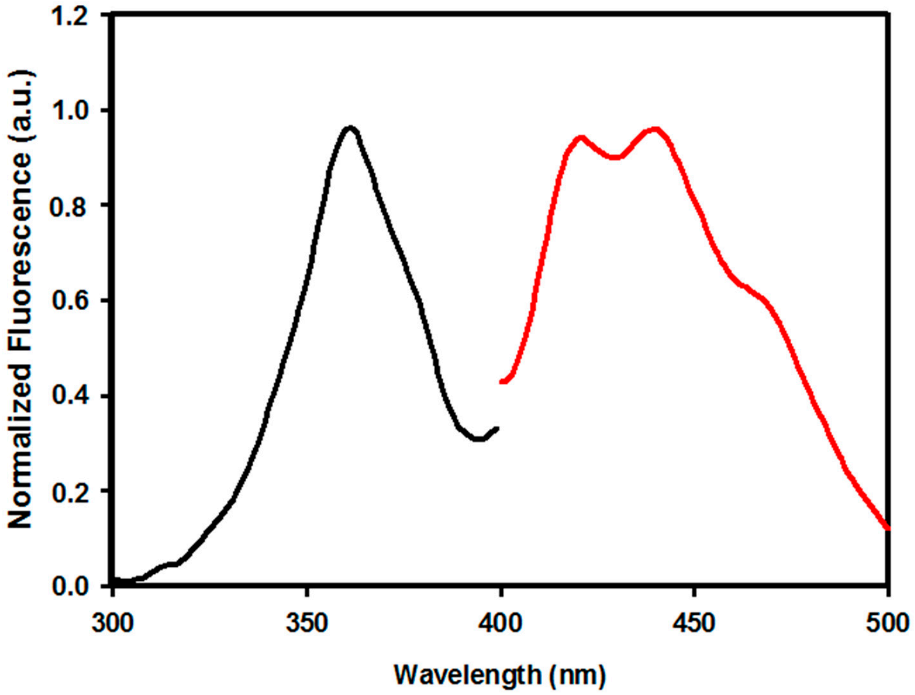

Figure S28. Excitation (black) and emission (red) spectra of derivative **3e** at concentrations of 15 μM in Tris-HCl buffer. Excitation at 361 nm and emission at 441 nm.

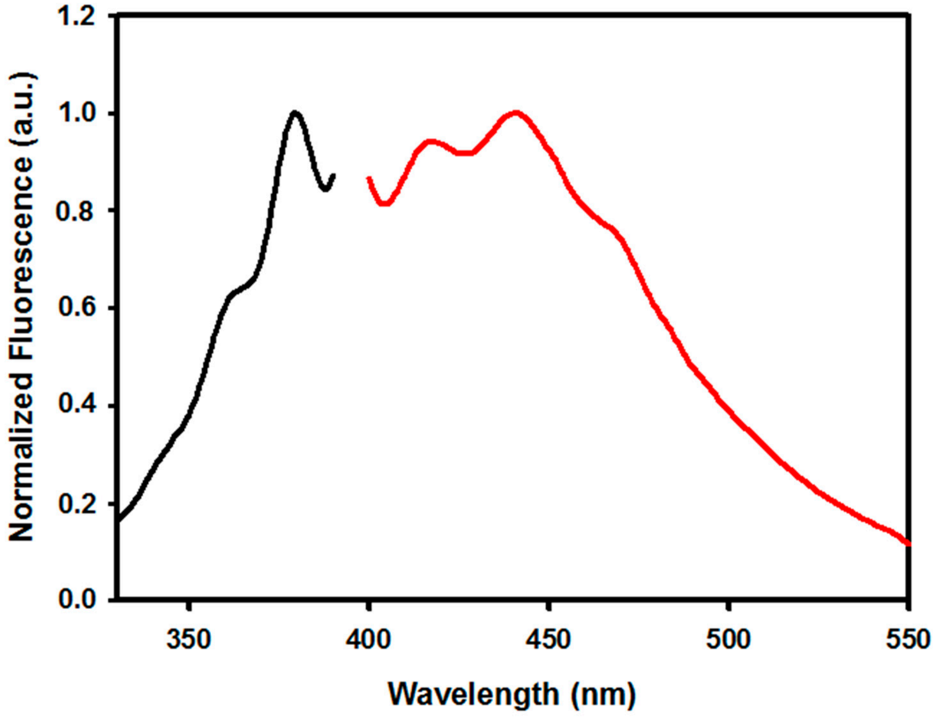

Figure S29. Excitation (black) and emission (red) spectra of derivative **3f** at concentrations of 15 µM in Tris-HCl buffer. Excitation at 355 nm and emission at 440 nm.

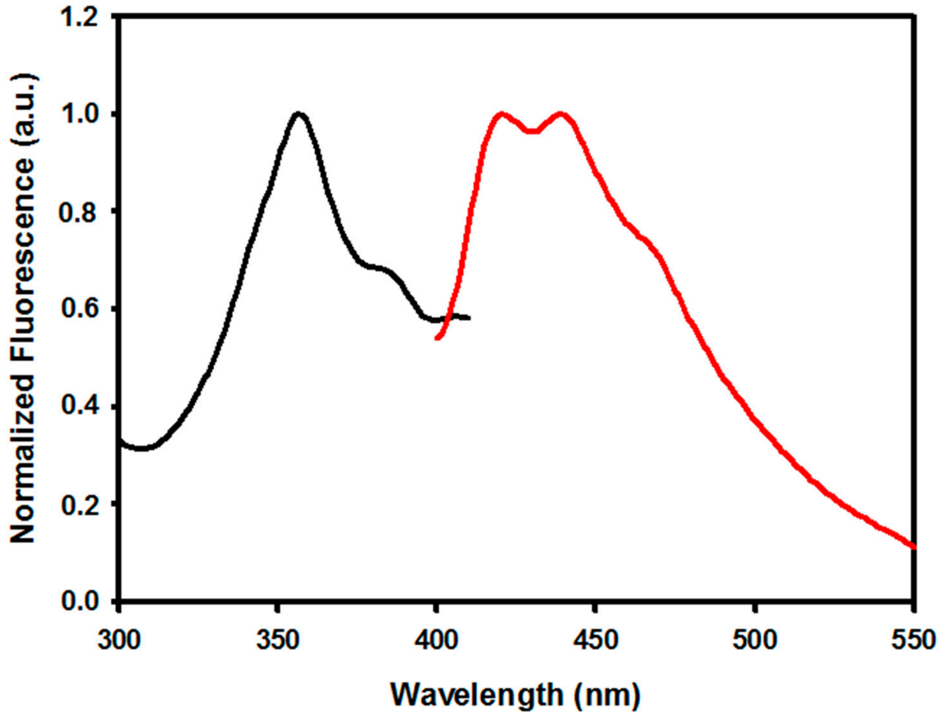

Figure S30. Excitation (black) and emission (red) spectra of derivative **3g** at concentrations of 15 µM in Tris-HCl buffer. Excitation at 352 nm and emission at 439 nm.

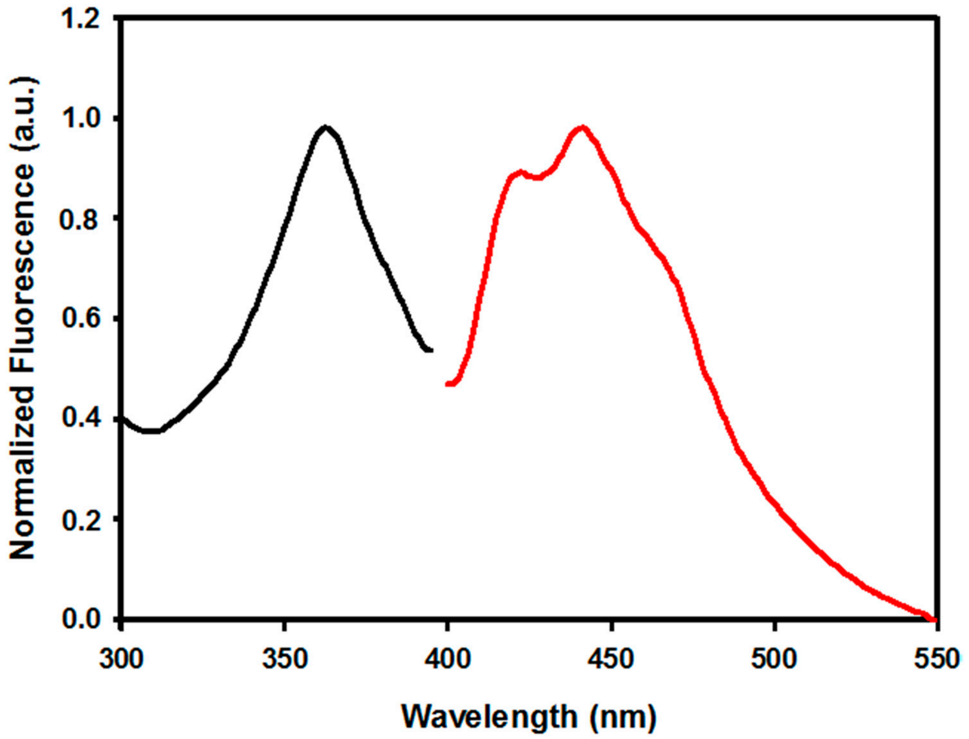

Figure S31. Excitation (black) and emission (red) spectra of derivative **3h** at concentrations of 15 µM in Tris-HCl buffer. Excitation at 350 nm and emission at 439 nm.

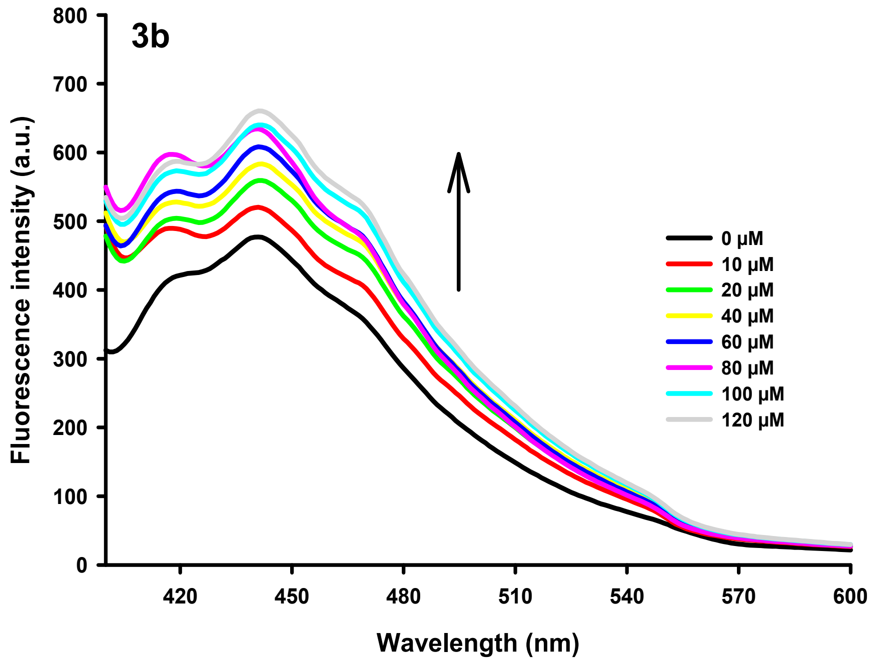

Figure S32. Fluorescence spectra of derivative **3b** (15 µM) with increasing concentrations of ctDNA. [DNA] = 0 (gray), 0 (black), 20 (red), 40 (green), 60 (yellow), 80 (dark blue), 100 (pink) and 120 (light blue). Arrow (↑) refers to hyperchromic effect.

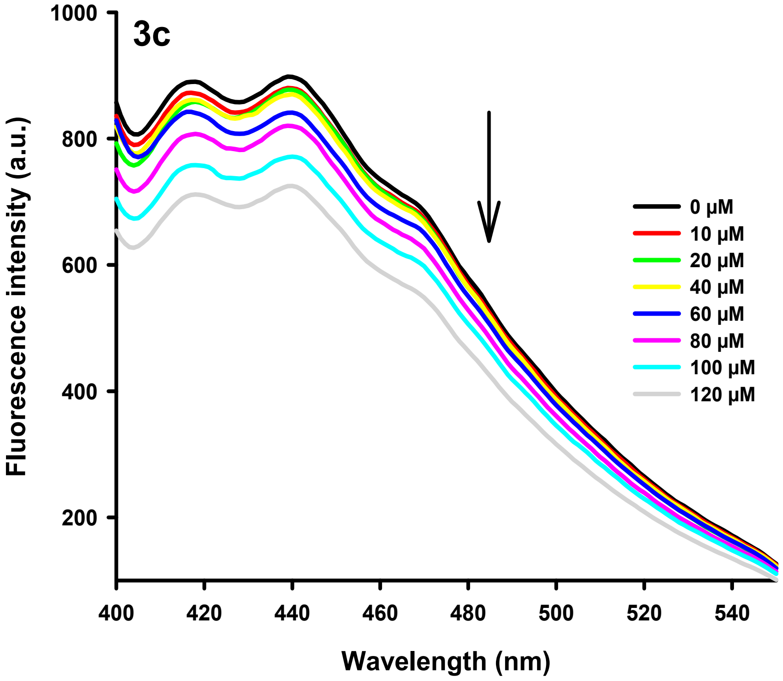

Figure S33. Fluorescence spectra of derivative **3c** (15 µM) with increasing concentrations of ctDNA. [DNA] = 0 (gray), 0 (black), 20 (red), 40 (green), 60 (yellow), 80 (dark blue), 100 (pink) and 120 (light blue). Arrow (↓) refers to hypochromic effect.

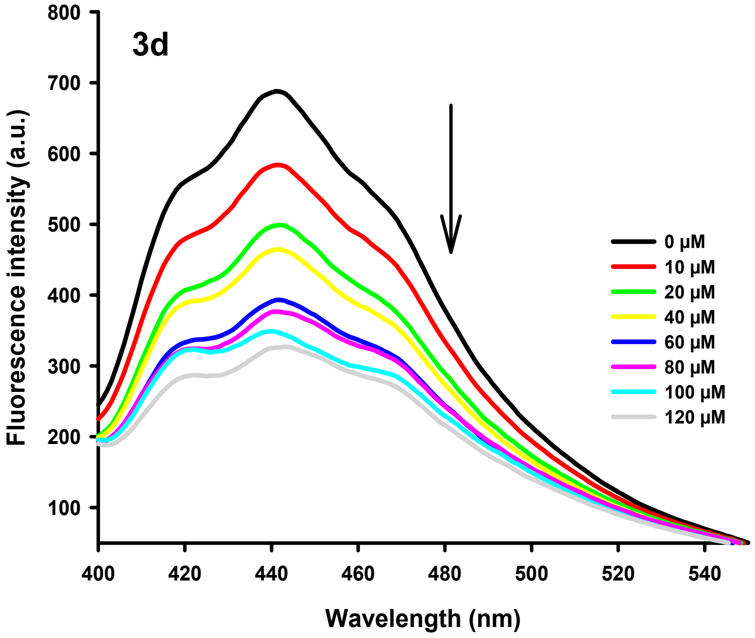

Figure S34. Fluorescence spectra of derivative **3d** (15 µM) with increasing concentrations of ctDNA. [DNA] = 0 (gray), 0 (black), 20 (red), 40 (green), 60 (yellow), 80 (dark blue), 100 (pink) and 120 (light blue). Arrow (↓) refers to hypochromic effect.

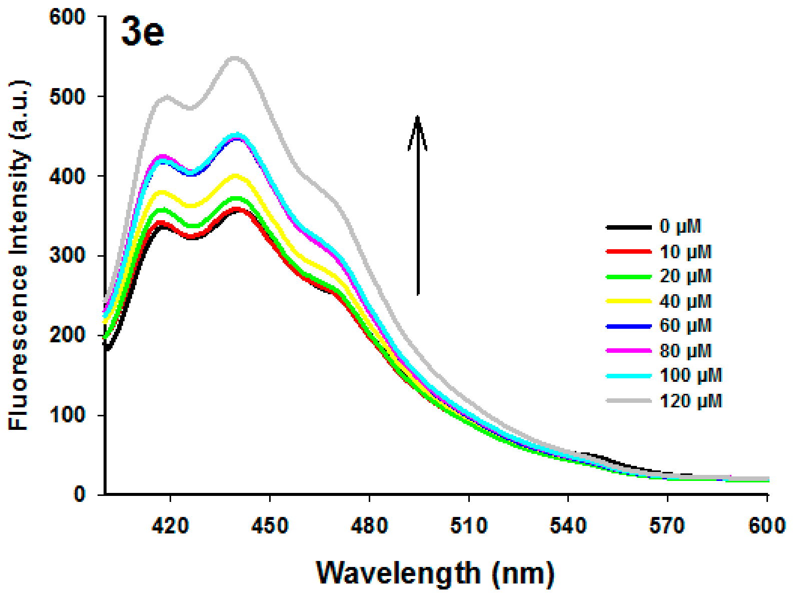

Figure S35. Fluorescence spectra of derivative **3e** (15 μM) with increasing concentrations of ctDNA. [DNA] = 0 (gray), 0 (black), 20 (red), 40 (green), 60 (yellow), 80 (dark blue), 100 (pink) and 120 (light blue). Arrow (↑) refers to hyperchromic effect.

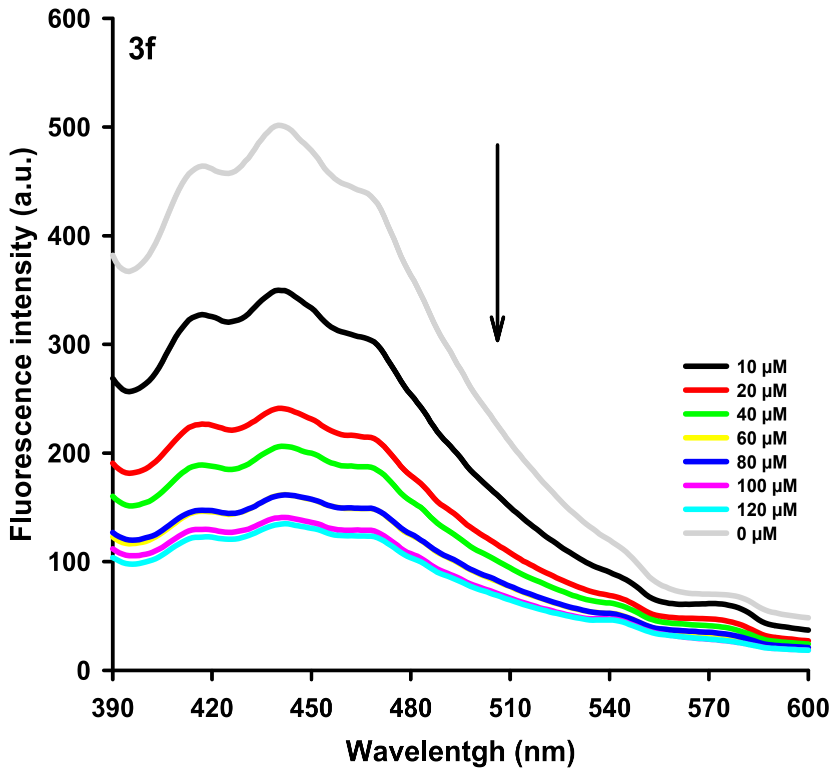

Figure S36. Fluorescence spectra of derivative **3f** (15 μM) with increasing concentrations of ctDNA. [DNA] = 0 (gray), 0 (black), 20 (red), 40 (green), 60 (yellow), 80 (dark blue), 100 (pink) and 120 (light blue). Arrow (↓) refers to hypochromic effect.

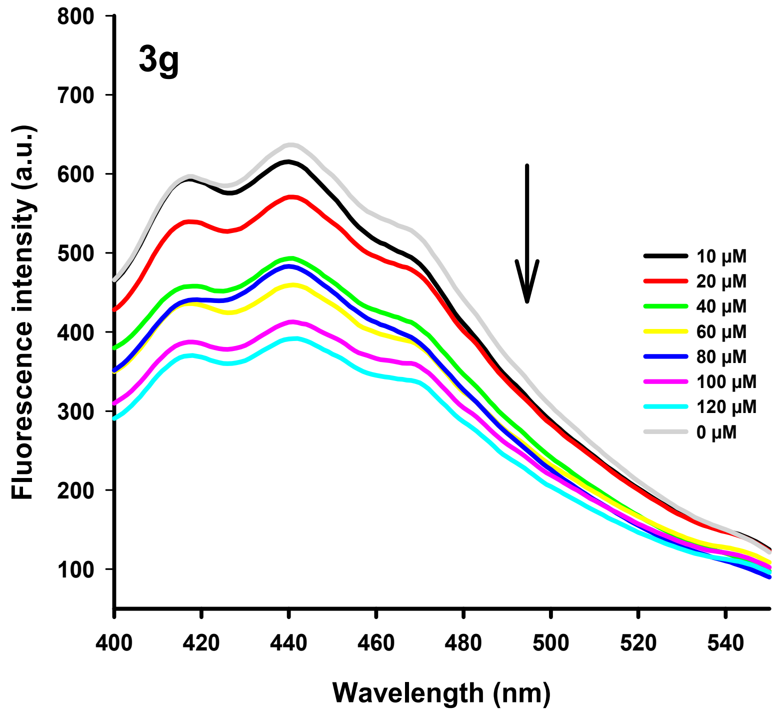

Figure S37. Fluorescence spectra of derivative **3g** (15 µM) with increasing concentrations of ctDNA. [DNA] = 0 (gray), 0 (black), 20 (red), 40 (green), 60 (yellow), 80 (dark blue), 100 (pink) and 120 (light blue). Arrow (↓) refers to hypochromic effect.

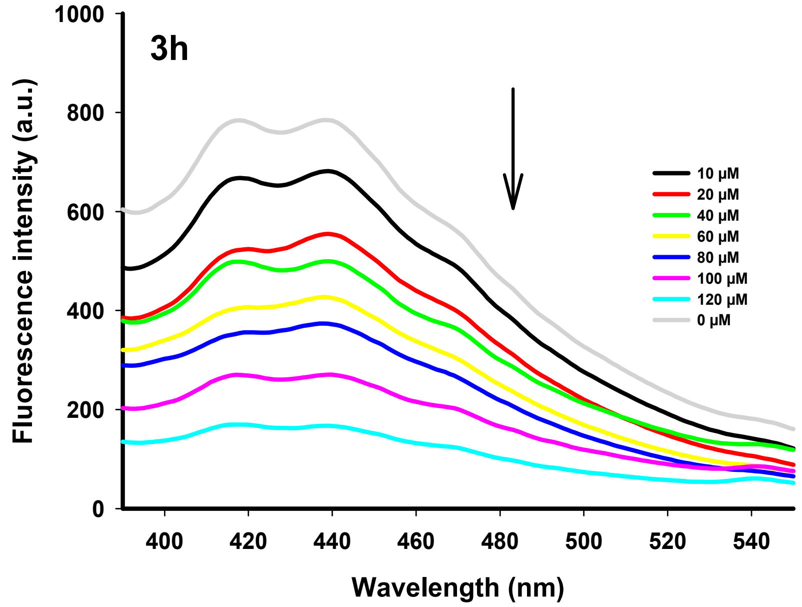

Figure S38. Fluorescence spectra of derivative **3h** (15 µM) with increasing concentrations of ctDNA. [DNA] = 0 (gray), 0 (black), 20 (red), 40 (green), 60 (yellow), 80 (dark blue), 100 (pink) and 120 (light blue). Arrow (↓) refers to hypochromic effect.

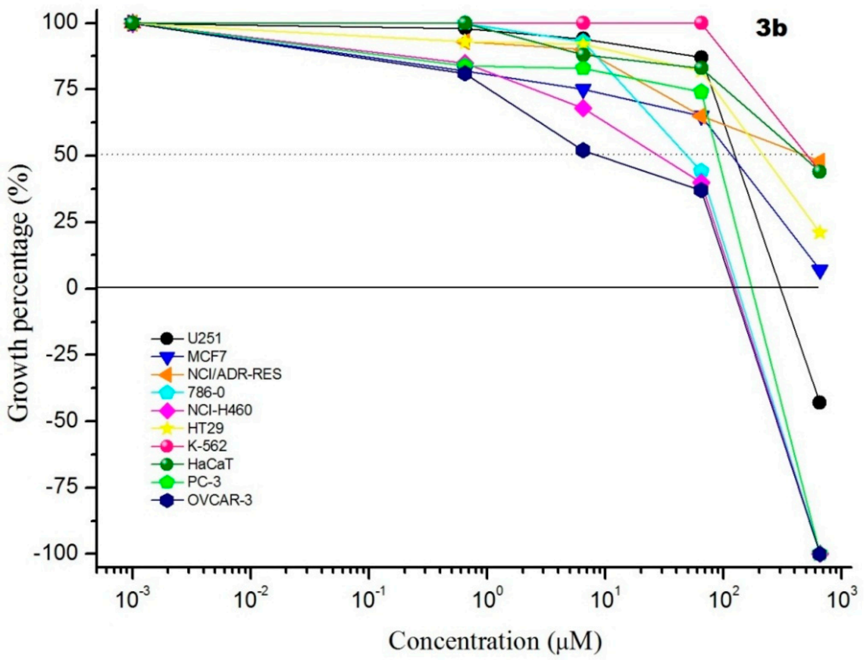

Figure S39. Antiproliferative activity of **3b** against nine cancerous cell lines: U251 (glioma, SNC); MCF-7 (breast adenocarcinoma); NCI-ADR/RES (ovary, multidrug resistance phenotype); 786-0 (kidney); NCI-H460 (lung non-small cell adenocarcinoma); PC-3 (prostate); OVCAR-3 (ovary); HT-29 (colon); K-562 (Chronic myeloid leukemia) and human keratinocytes (HaCaT).

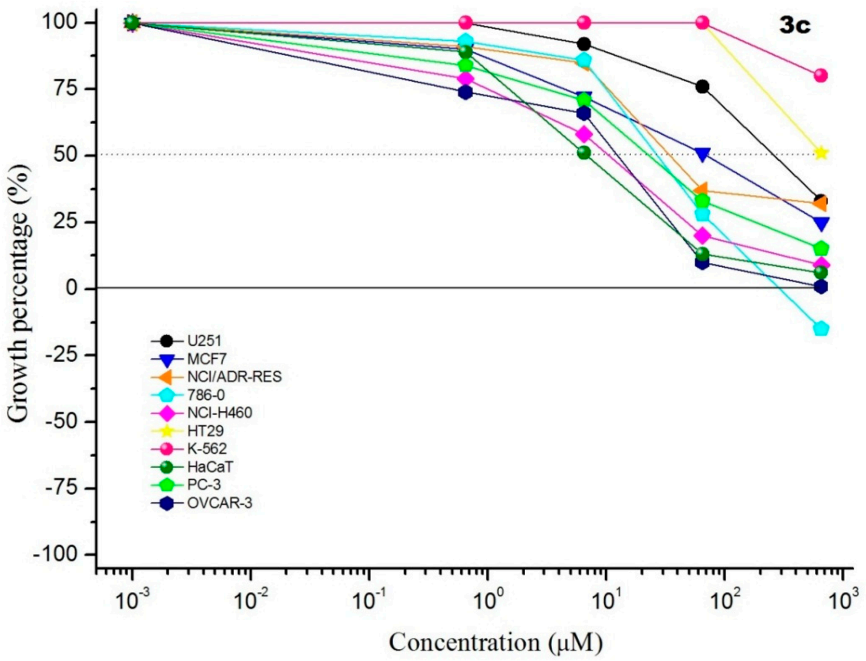

Figure S40. Antiproliferative activity of **3c** against nine cancerous cell lines: U251 (glioma, SNC); MCF-7 (breast adenocarcinoma); NCI-ADR/RES (ovary, multidrug resistance phenotype); 786-0 (kidney); NCI-H460 (lung non-small cell adenocarcinoma); PC-3 (prostate); OVCAR-3 (ovary); HT-29 (colon); K-562 (Chronic myeloid leukemia) and human keratinocytes (HaCaT).

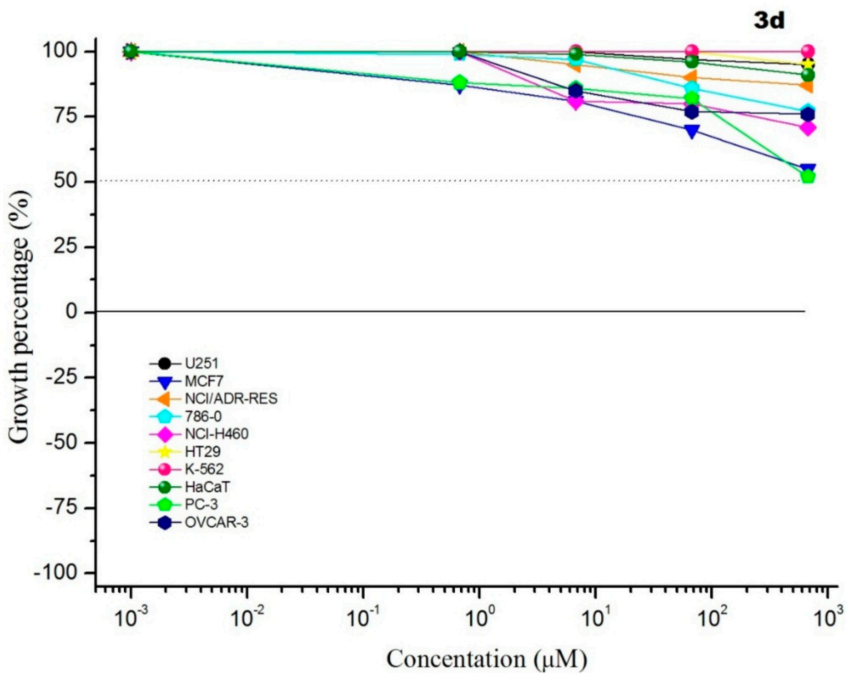

Figure S41. Antiproliferative activity of **3d** against nine cancerous cell lines: U251 (glioma, SNC); MCF-7 (breast adenocarcinoma); NCI-ADR/RES (ovary, multidrug resistance phenotype); 786-0 (kidney); NCI-H460 (lung non-small cell adenocarcinoma); PC-3 (prostate); OVCAR-3 (ovary); HT-29 (colon); K-562 (Chronic myeloid leukemia) and human keratinocytes (HaCaT).

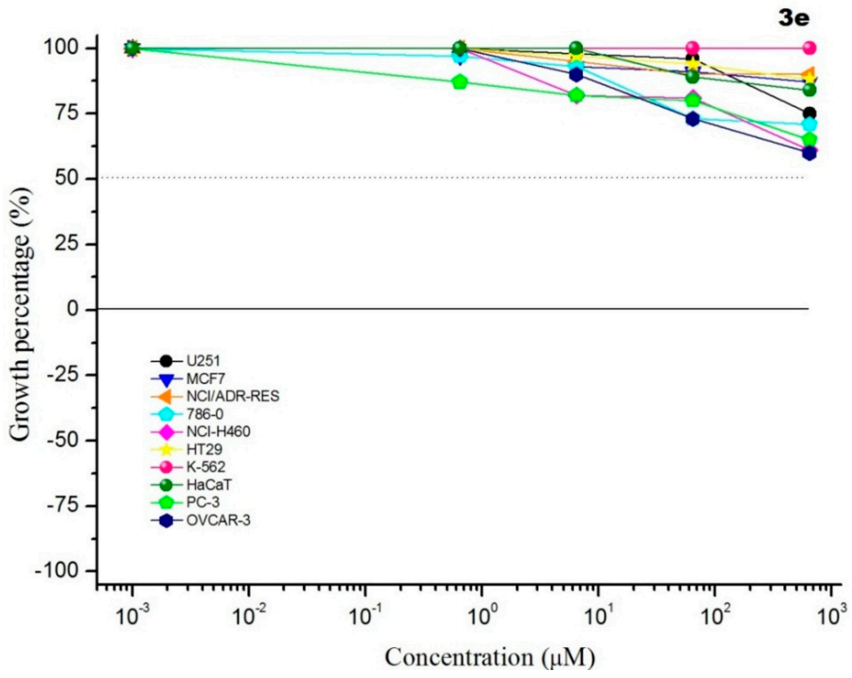

Figure S42. Antiproliferative activity of **3e** against nine cancerous cell lines: U251 (glioma, SNC); MCF-7 (breast adenocarcinoma); NCI-ADR/RES (ovary, multidrug resistance phenotype); 786-0 (kidney); NCI-H460 (lung non-small cell adenocarcinoma); PC-3 (prostate); OVCAR-3 (ovary); HT-29 (colon); K-562 (Chronic myeloid leukemia) and human keratinocytes (HaCaT).

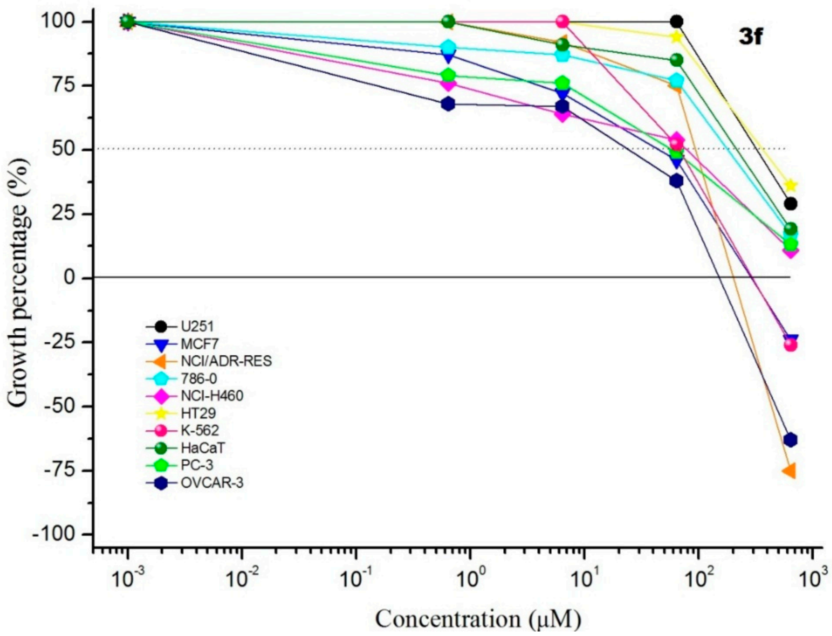

Figure S43. Antiproliferative activity of **3f** against nine cancerous cell lines: U251 (glioma, SNC); MCF-7 (breast adenocarcinoma); NCI-ADR/RES (ovary, multidrug resistance phenotype); 786-0 (kidney); NCI-H460 (lung non-small cell adenocarcinoma); PC-3 (prostate); OVCAR-3 (ovary); HT-29 (colon); K-562 (Chronic myeloid leukemia) and human keratinocytes (HaCaT).

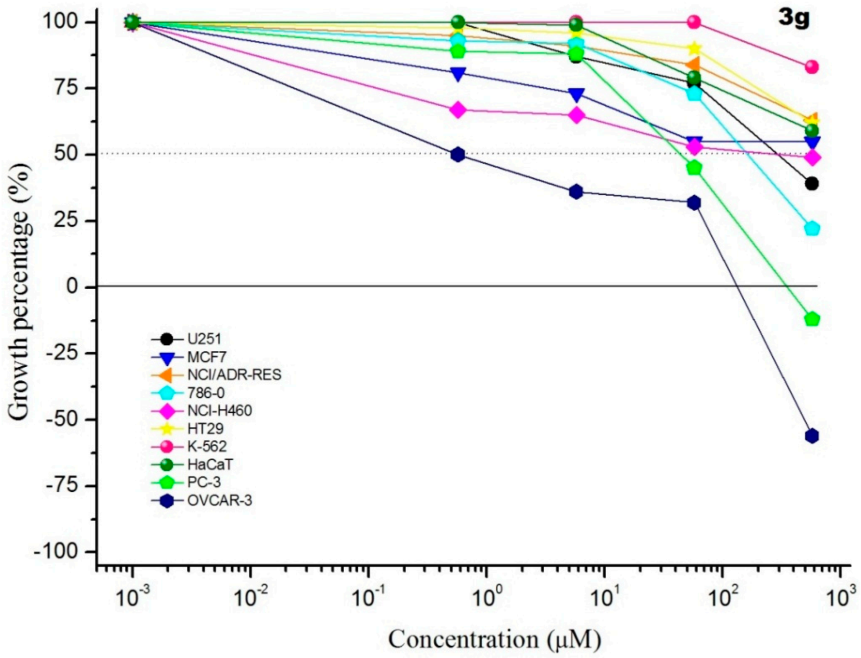

Figure S44. Antiproliferative activity of **3g** against nine cancerous cell lines: U251 (glioma, SNC); MCF-7 (breast adenocarcinoma); NCI-ADR/RES (ovary, multidrug resistance phenotype); 786-0 (kidney); NCI-H460 (lung non-small cell adenocarcinoma); PC-3 (prostate); OVCAR-3 (ovary); HT-29 (colon); K-562 (Chronic myeloid leukemia) and human keratinocytes (HaCaT).

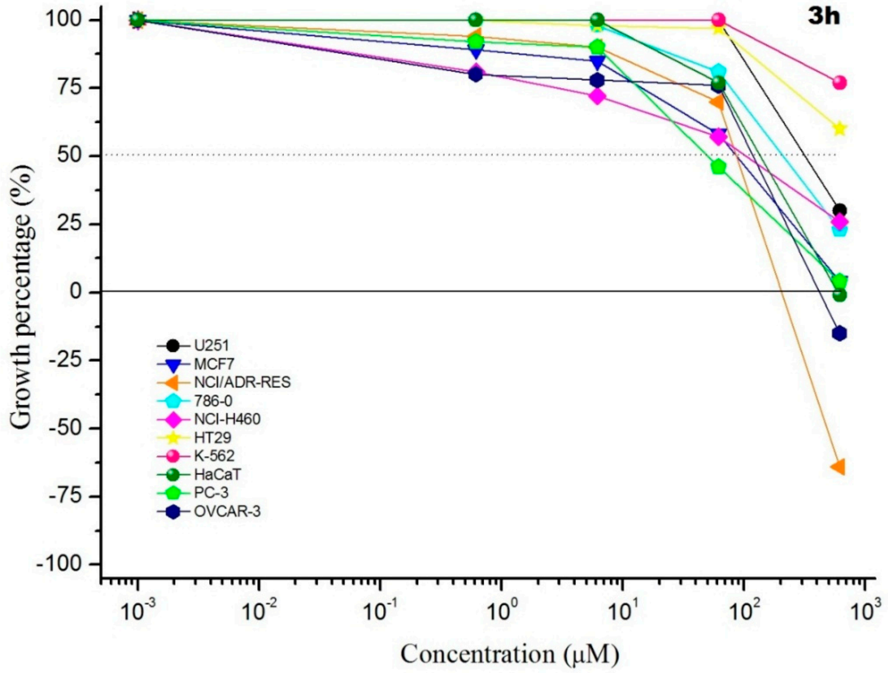

Figure S45. Antiproliferative activity of **3h** against nine cancerous cell lines: U251 (glioma, SNC); MCF-7 (breast adenocarcinoma); NCI-ADR/RES (ovary, multidrug resistance phenotype); 786-0 (kidney); NCI-H460 (lung non-small cell adenocarcinoma); PC-3 (prostate); OVCAR-3 (ovary); HT-29 (colon); K-562 (Chronic myeloid leukemia) and human keratinocytes (HaCaT).

Table S1. Cell lines used *in vitro* antiproliferative assays and their inoculation densities.

Cell Lines	Density (×10^4 cells/mL)
U251 (glioma, SNC)	4.0
MCF-7 (breast adenocarcinoma)	6.0
NCI-ADR/RES (ovary, multidrug resistance phenotype)	5.0
786-O (kidney)	4.5
NCI-H460 (lung non-small cell adenocarcinoma)	4.0
PC-3 (prostate)	4.0
OVCAR-3 (ovary)	7.0
HT-29 (colon)	4.0
K-562 (Chronic myeloid leukemia)	6.0
HaCaT (human keratinocytes)	4.0

Table S2. Exact mass, calculated and found *m*/*z* values for compounds (**3a**–**h**).

Compound	Exact Mass Calculated *	Calculated *m*/*z* **	Found [M + 1] **
**3a**	356.1096	356.1096 (100.0%), 357.1129 (22.7%), 358.1054 (4.5%), 358.1163 (2.5%), 357.1066 (1.5%), 359.1087 (1.0%)	357.124
**3b**	384.1409	384.1409 (100.0%), 385.1442 (24.9%), 386.1367 (4.5%), 386.1476 (3.0%), 385.1379 (1.5%), 387.1400 (1.1%)	385.134
**3c**	384.1409	384.1409 (100.0%), 385.1442 (24.9%), 386.1367 (4.5%), 386.1476 (3.0%), 385.1379 (1.5%), 387.1400 (1.1%)	385.131
**3d**	370.1252	370.1252 (100.0%), 371.1286 (23.8%), 372.1210 (4.5%), 372.1319 (2.7%), 371.1223 (1.5%), 373.1224 (1.1%)	371.120
**3e**	386.1201	386.1201 (100.0%), 387.1235 (23.8%), 388.1159 (4.5%), 388.1268 (2.7%), 387.1172 (1.5%), 389.1193 (1.1%)	387.073
**3f**	390.0706	390.0706 (100.0%), 392.0676 (32.0%), 391.0739 (22.7%), 393.0710 (7.3%), 392.0664 (4.5%), 392.0773 (2.5%), 391.0676 (1.5%), 394.0634 (1.4%), 393.0697 (1.0%)	391.041
**3g**	434.0201	434.0201 (100.0%), 436.0180 (97.3%), 435.0234 (22.7%), 437.0214 (22.1%), 436.0159 (4.5%), 438.0138 (4.4%), 436.0268 (2.5 %), 438.0247 (2.4%), 435.0107 (1.5%), 437.0151 (1.4%), 437.0192 (1.0%)	436.968
**3h**	406.1252	406.1252 (100.0%), 407.1286 (27.0%), 408.1210 (4.5%), 408.1319 (3.5%), 407.1223 (1.5%), 409.1244 (1.2%)	407.024

* The values were calculated using the software ChemDraw 12 (PerkinElmer Informatics, Waltham, MA, USA); ** The values corresponding to found molecular ions mass and match to the secondary calculated *m*/*z* peaks. Mass spectra were recorded on matrix-assisted laser desorption/ionization recorded with a time-of-flight mass spectrometer (MALDI-TOF).
